# Latent Regulatory Potential of Human-Specific Repetitive Elements

**DOI:** 10.1016/j.molcel.2012.11.013

**Published:** 2013-01-24

**Authors:** Michelle C. Ward, Michael D. Wilson, Nuno L. Barbosa-Morais, Dominic Schmidt, Rory Stark, Qun Pan, Petra C. Schwalie, Suraj Menon, Margus Lukk, Stephen Watt, David Thybert, Claudia Kutter, Kristina Kirschner, Paul Flicek, Benjamin J. Blencowe, Duncan T. Odom

**Affiliations:** 1 University of Cambridge, Cancer Research UK-Cambridge Institute, Robinson Way, Cambridge CB2 0RE, UK; 2 Banting and Best Department of Medical Research and Department of Molecular Genetics, Donnelly Centre, Toronto, ON M5S 3E1, Canada; 3 European Bioinformatics Institute (EMBL-EBI), Wellcome Trust Genome Campus, Hinxton CB10 1SD, UK; 4 Wellcome Trust Sanger Institute, Wellcome Trust Genome Campus, Hinxton CB10 1SA, UK

## Abstract

At least half of the human genome is derived from repetitive elements, which are often lineage specific and silenced by a variety of genetic and epigenetic mechanisms. Using a transchromosomic mouse strain that transmits an almost complete single copy of human chromosome 21 via the female germline, we show that a heterologous regulatory environment can transcriptionally activate transposon-derived human regulatory regions. In the mouse nucleus, hundreds of locations on human chromosome 21 newly associate with activating histone modifications in both somatic and germline tissues, and influence the gene expression of nearby transcripts. These regions are enriched with primate and human lineage-specific transposable elements, and their activation corresponds to changes in DNA methylation at CpG dinucleotides. This study reveals the latent regulatory potential of the repetitive human genome and illustrates the species specificity of mechanisms that control it.

## Highlights

► A mouse carrying human chromosome 21 fails to repress primate-specific repeats ► The lack of repression was revealed by H3K4me3 and transcription factor binding ► Activation corresponded to a decrease in CpG methylation ► Primate-specific repeats activated in human testes were activated in the Tc1 mouse

## Introduction

Between one-half and possibly up to two-thirds of the human genome is derived from repetitive sequences, most of which are classified as transposable elements (TEs) (de Koning et al., 2011). TEs can serve as regulatory DNA contributing to tissue-specific transcriptional evolution (Bourque et al., 2008; Faulkner et al., 2009; Lowe et al., 2007; Oliver and Greene, 2009), and their activity has altered the regulatory circuitry of embryonic stem cells (Kunarso et al., 2010), mammalian pregnancy pathways (Lynch et al., 2011; Xie et al., 2010), and the deployment of CTCF binding sites across mammalian genomes (Bourque et al., 2008; Schmidt et al., 2012). The rapid increase in sequenced mammalian genomes (Lindblad-Toh et al., 2011), in vivo multivertebrate transcription factor binding maps (Kunarso et al., 2010; Schmidt et al., 2010, 2012), and computational tools to dissect repeat-based genomes (Treangen and Salzberg, 2012) has uncovered lineage-specific genome innovations whose biological functions are not known.

Relative to the mobility of TEs in species such as maize, *Drosophila*, mice, and some primates (Maksakova et al., 2006), the activity of retrotransposons has declined in hominids (Lander et al., 2001), yet TEs continue to shape the human genome. The insertion of TEs underlies at least 65 human diseases (Cordaux and Batzer, 2009; Goodier and Kazazian, 2008) and can provide a substrate for nonallelic homologous recombination, resulting in structural changes (Beck et al., 2011). As transposons rapidly acquire mutations and as their activity can damage a genome, multiple mechanisms have evolved to silence them in mammals (Levin and Moran, 2011), including specific histone modifications, DNA methylation, and targeted small RNAs (De Fazio et al., 2011; Maksakova et al., 2008; Rebollo et al., 2011; Reuter et al., 2011).

One consequence of the arms race between transposons and transposon silencing mechanisms is that the regulatory potential of a transposon-derived sequence is difficult to evaluate in its host which has coevolved mechanisms to repress it. Thus, functional in vivo studies of TEs often employ heterologous strategies such as placing human retroelements into other vertebrate species including mouse, rat (Kano et al., 2009), and zebrafish (Pi et al., 2004). Using an aneuploid mouse that stably transmits a majority (42 of 46.9 Mb) of human chromosome 21 (HsChr21) through the female germline (Tc1) (O’Doherty et al., 2005), we previously demonstrated that the nonrepetitive portion of mammalian genomes is largely transcriptionally directed by *cis*-acting regulatory elements (Wilson et al., 2008). This study employed genome-tiling microarrays, which by design did not permit the analysis of repetitive regions. Using high-throughput sequencing, which allows us to explore a greater percentage of the TE-derived genome, we here explore the in vivo transcriptional control of a human chromosome placed in a heterologous mouse environment. This unique system revealed unexpected regulatory and transcriptional potential in many recently evolved human sequences which are associated with changes in DNA methylation and chromatin state.

## Results

### Many HsChr21 Regions Transcriptionally Active in Tc1 Mouse Tissues Are Silent in Human

**Figure 1:**
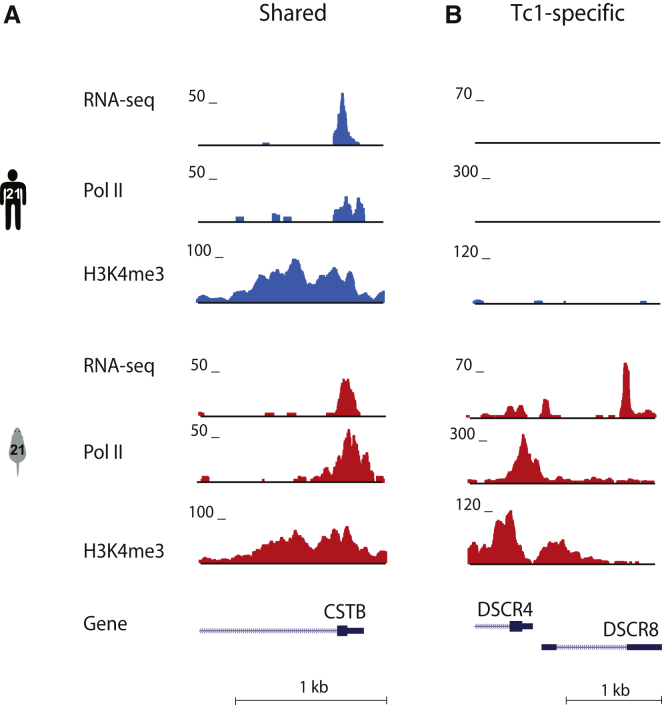
In a Mouse Carrying Human Chromosome 21, Most Locations of Human Chromosome 21 Are Transcribed in Liver Largely as in Human Tissues, Yet Specific Loci Show Differences (A) High-throughput sequencing of chromatin immunoprecipitations and poly(A) mRNA enrichment revealed that the *CSTB* locus is occupied by RNA polymerase II, enriched for H3K4me3, and transcribed into RNA in both human and Tc1 mouse liver. (B) The *DSCR4/8* locus on HsChr21 shows similar evidence of transcription in Tc1 mouse liver tissue, which is not evident in normal human liver.

To compare in vivo gene regulation of both repetitive and nonrepetitive regions of HsChr21 between human and Tc1 mouse livers, we experimentally profiled the following using high-throughput sequencing methodologies: (1) polyadenylated (poly[A])-containing mRNA transcripts, (2) regions enriched for trimethylation of H3K4 (H3K4me3) as a marker for transcriptional initiation (Bernstein et al., 2005; Guenther et al., 2007; Heintzman et al., 2007), and (3) genomic occupancy of the RNA polymerase II (Pol II) basal machinery (Figure 1; Experimental Procedures; see Table S1A, Document S2, and Figure S1 online).

Using H3K4me3 as a proxy for transcription initiation, we found that most regions activated on HsChr21 (214/383) were not significantly different in quality and quantity between the two species (we henceforth refer to these sites as Shared; Figure 1A, Figure S2A, Experimental Procedures). By using sequencing-based methods instead of microarrays, we also identified specific human sequence locations uniquely activated in the mouse nucleus. For example, in Tc1 mouse livers, the bidirectional promoter of the Down’s Syndrome critical regions 4 and 8 (*DSCR4* and *DSCR8*) is bound by Pol II, enriched for H3K4me3, and generates poly(A) transcripts (Figure 1B). Normally, these two long noncoding RNAs are transcriptionally driven in human placenta by a primate-specific LTR retrotransposon but are silenced in liver (Dunn et al., 2006).

**Figure 2:**
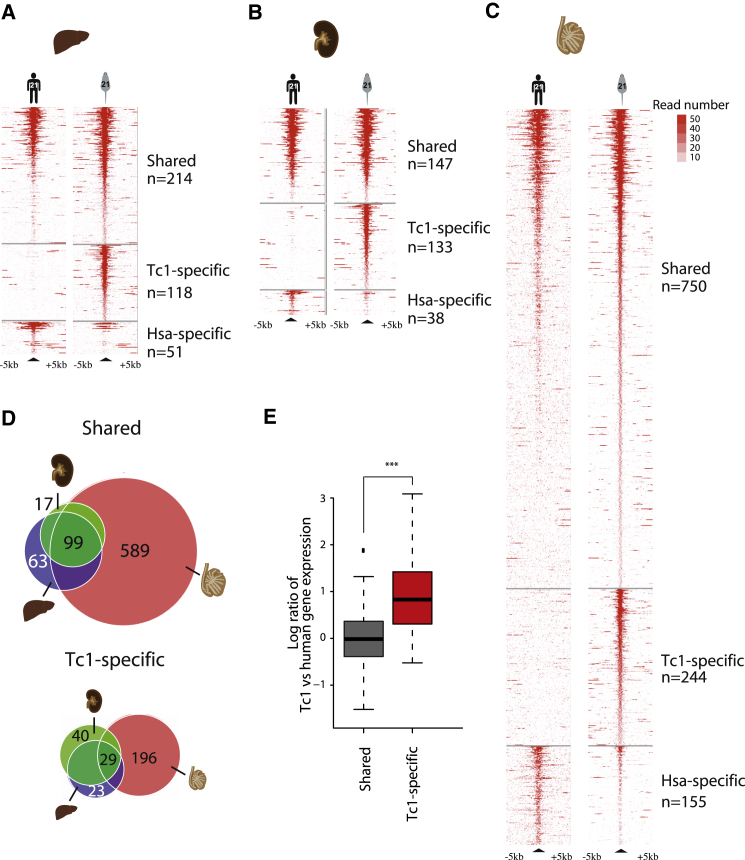
Tc1-Specific Transcription Initiation Occurs in Somatic and Germline Tissues and Influences Gene Expression (A–C) Chromosome-wide identification of all regions enriched for H3K4me3 on HsChr21 in mouse and human liver, kidney, and testes; heatmap is sorted by descending signal strength and by species specificity. (D) Similar sets of loci on HsChr21 are enriched for H3K4me3 in both mouse and human, and these are largely a subset of loci found in testes in both species. In liver and kidney, Tc1-specific loci enriched for H3K4me3 were found in similar locations, which were distinct from those found in Tc1 mouse testes. (E) H3K4me3-enriched sites in the Shared and Tc1-specific categories in liver were associated with the nearest transcription start site and the expression levels of genes on Tc1-HsChr21 determined relative to human genes (Shared, n = 86; Tc1 specific, n = 17) (^∗∗∗^p ≤ 0.0005, one-sided Mann-Whitney U test).

In total, we identified 118 regions on HsChr21 enriched for trimethylation of H3K4 in Tc1 mouse liver compared to human (henceforth Tc1-specific), which we defined as having at least 4-fold greater normalized read counts, with an FDR of <0.1 (Experimental Procedures, Figure 2A, Figures S2A and S2B). We confirmed that the Tc1-specific regions were robust and could not be explained by misalignment of sequencing reads with control experiments including paired-end mapping of chromatin immunoprecipitation (ChIP) experiments (Table S1A), different alignment strategies (Tables S1B–S1D, Figure S2C), and were also validated by qPCR (Table S1E and Figure S2D).

Only 51 locations on HsChr21 activated in human liver were found enriched relative to Tc1 mouse liver (henceforth Human-specific) using these criteria. Notably, in contrast to most Tc1-specific activated regions, which largely appeared unique to the mouse, regions identified as containing H3K4me3 specifically in human often showed measurable signal in the Tc1 mouse (Figure 2, Figures S2E and S2F).

The design and sensitivity of genome tiling microarrays used by the prior study that compared human and Tc1 mouse liver gene regulation (Wilson et al., 2008) prevented the identification of the majority of these Tc1-specific regions, discovered here by ChIP-seq. Because repetitive sequences are excluded from microarray design (Bertone et al., 2006), 40% of the Tc1-specific regions (n = 47) overlapped less than three microarray probes, which was the minimum criteria to identify regions bound by ChIP experiments. Indeed, only 907 of the 74,901 chromosome 21 probes on the Agilent microarray overlap with sequences in the RepeatMasker library. Thirty-seven percent (n = 44) of the Tc1-specific regions that overlapped three or more probes had insufficient signal to be called in either species in our previous study. Of the remaining 27 Tc1-specific H3K4me3 regions that were identified on microarrays, only nine were classified as unique to the Tc1 mouse. In summary, our current study validates over 94% of the Shared H3K4me3 regions originally identified by ChIP-chip; however, due to the increased coverage and sensitivity afforded by sequencing, we newly identify Tc1-specific genomic regions associated with activated chromatin and repetitive elements.

### Consequences of Tc1-Specific Transcriptional Activation

H3K4me3 has been shown to serve as an anchorage for the basal transcriptional machinery (Bernstein et al., 2005; Guenther et al., 2007; Heintzman et al., 2007; Vermeulen et al., 2007). We looked for evidence for transcriptional activation near HsChr21 regions associated with H3K4me3. Tc1-specific H3K4me3 regions both have higher overlap with Pol II in Tc1 mouse liver than in human liver (74% versus 28%, respectively; Figure S2G) and are located near genes with higher gene expression in the Tc1 mouse (Figure 2 and Figure S2G). Many Tc1-specific regions have therefore resulted in changes in gene expression, highlighting their latent regulatory potential.

We asked whether the transcriptional activation observed in Tc1 mouse liver also occurred in other somatic tissues. We mapped H3K4me3-associated DNA regions in Tc1 mouse and human kidney samples and identified a similar set of Tc1-specific transcriptionally active regions, many of which were shared with liver (Figure 2B and Table S3). H3K4me3 profiling in Tc1 mouse brain, spleen, and muscle tissues revealed that at least 50% of Tc1-specific liver regions are enriched across all profiled somatic tissues (Table S3 and Figure S2H). Thus, the occurrence of Tc1-specific H3K4me3 regions on HsChr21 occurs broadly across somatic mouse tissues.

Relative to somatic tissues, testes transcribe a greater proportion of the mouse genome (Shima et al., 2004), and this transcription is accompanied by an increased number of H3K4me3-associated regions (Smagulova et al., 2011). We tested the possibility that this global transcriptional upregulation in germline tissues might further unmask additional latent regulatory regions in the human genome. Consistent with these reports, more than twice as many H3K4me3-associated regions were identified on HsChr21 in testes from either Tc1 mouse (n = 994) or human (n = 905), compared to somatic tissues (Figure 2C). In testes, the majority of regions enriched in H3K4me3 were shared between human and mouse (n = 750) and appear to be a superset encompassing those found in liver and kidney (Figure 2D). The regions enriched for H3K4me3 specifically in Tc1 mouse testes were largely distinct from those found specifically in Tc1 mouse liver and kidney (Figure 2D and Table S3). In sum, our data indicate that latent regulatory regions in the human genome can become transcriptionally activated in somatic and germline tissues of a heterologous species.

### Young, Primate-Specific Repeats Can Be Transcriptionally Activated in the Tc1 Mouse

**Figure 3:**
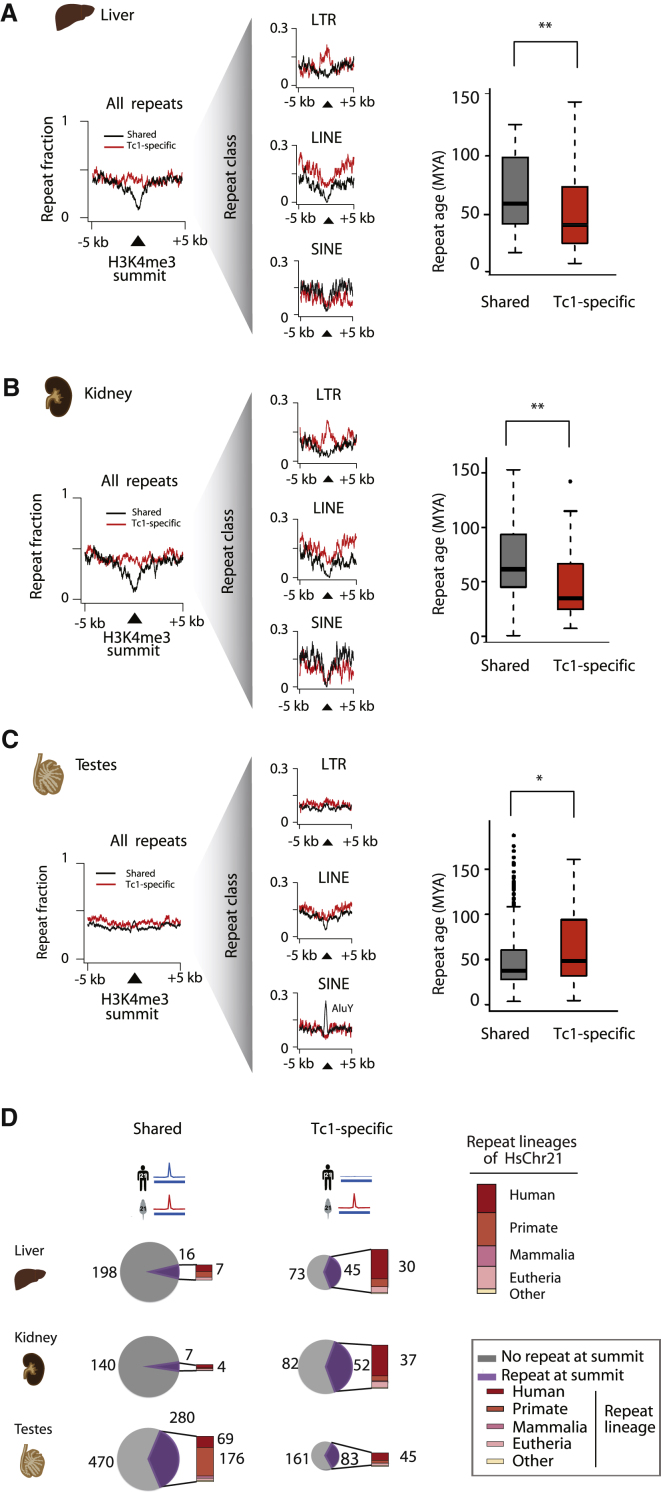
Tc1-Specific Locations of Transcription Initiation Are Enriched for Young, Lineage-Specific Repetitive Elements (A) Fraction of repeat elements within a 10 kb window around the H3K4me3 peak summit in Shared (black) and Tc1-specific (red) events in liver. Shown is age of the repeats in Tc1-specific and Shared sites as determined by nucleotide substitution rates of repeat instances at H3K4me3 peak summits. (B) As in (A) for kidney. (C) As in (A) for testes. (D) Number and lineage of repeat elements at H3K4me3 peak summits in liver, kidney, and testes. Pie charts are scaled relative to the total number of H3K4me3 binding events in the Shared category where the purple proportion represents the fraction of H3K4me3 events that have a repeat element at the H3K4me3 peak summit. Bar charts represent the lineage in which the repeat elements originated; the numbers of human-specific (and primate-specific for Shared testes sites) are reported beside the bar charts. The composition of repeat lineages on HsChr21 is shown in the bar graph on the right panel (^∗^p ≤ 0.05, ^∗∗^p ≤ 0.005, Wilcoxon rank-sum test).

We asked whether the TE composition differed between the Tc1-specific H3K4me3 regions and those H3K4me3 regions that showed no significant differences between species. To do this, we collected all the repeat elements that were significantly enriched for H3K4me3 in liver, kidney, and testes and then subdivided them based on whether they were shared between species or unique to the Tc1 mouse (Figures 3A–3C). We found that repeat elements were significantly more likely to be specifically enriched for H3K4me3 in Tc1 liver and kidney tissues (p = 7.6 × 10^−9^ and 6.7 × 10^−9^, respectively) (Figures 3A and 3B, leftmost panels). In contrast to the somatic tissues, the testes do not show significant repetitive element enrichment between Tc1-specific and Shared H3K4me3 regions (p = 0.27; Tables S2A and S2B) (Figure 3C, leftmost panel).

To identify whether specific types of repeats were responsible for the transcriptional activation identified in the somatic tissues of the Tc1 mouse, we further subdivided the H3K4me3 regions by their component repeat class (Figures 3A–3C, middle panels). Most notably, LTR elements showed a clear, Tc1-specific enrichment of H3K4me3 in the liver and kidney (p < 10^−5^). Similarly, H3K4me3 in Tc1 mouse liver and kidney was enriched at LINE repeats, despite a more diffuse architecture (p < 10^−3^) (Figures 3A and 3B; Tables S2A and S2B, Figure S3A, Document S2). Five of the 20 human transposable SVA elements on chromosome 21 (Tc1-HsChr21) were significantly enriched for Tc1-specific H3K4me3 in liver (and 6/20 in kidney) (p < 0.03) (Table S2B).

In total, there are 41,877 repeat instances across Tc1-HsChr21, of which 1,043 were associated with H3K4me3. The distribution of the repeat classes captured in these H3K4me3 regions is similar to the repeat class distribution in the entire Tc1 HsChr21 chromosome (9% versus 8%, respectively, for DNA elements; 33% versus 22% for LINEs; 23% versus 25% LTRs; and 35% versus 43% for SINEs) (Tables S2C, Document S2). This suggests that the observed Tc1-specific repeat enrichments are not due to biases in repeat content in promoter regions.

**Figure 4:**
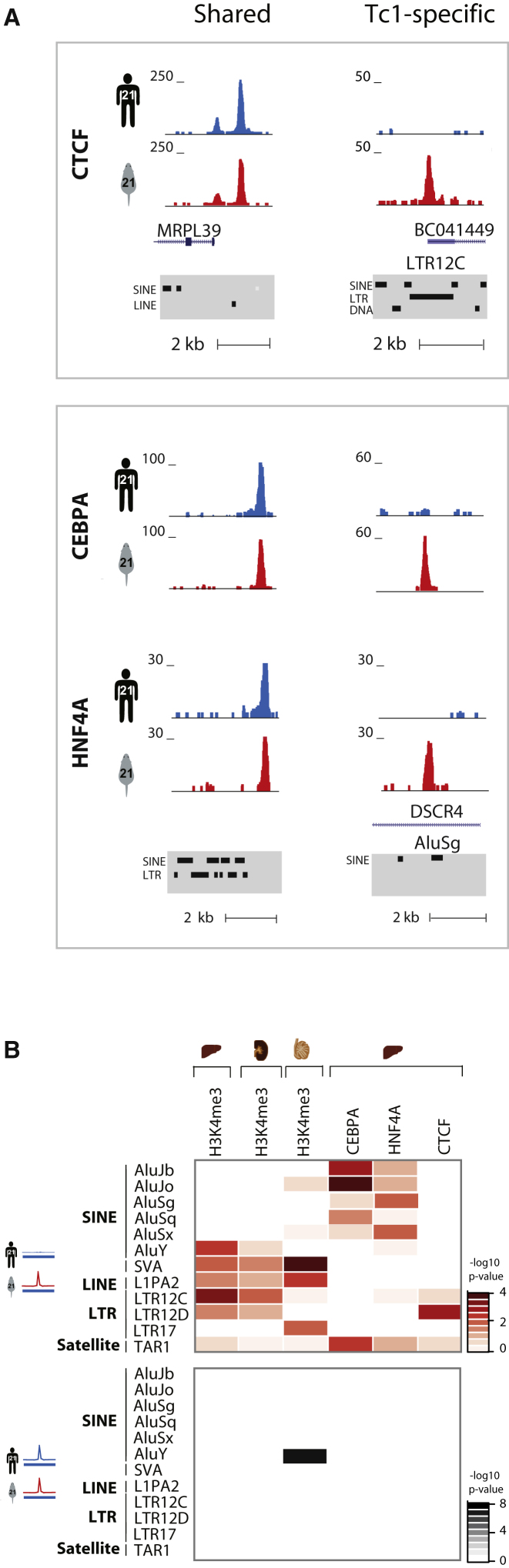
Repetitive Elements Contain Latent Transcriptional Regulator Binding Sites (A) Individual CEBPA, HNF4A, and CTCF binding sites can be carried by specific repeat elements. An LTR12C repetitive element upstream of the *SOD1* locus reveals a latent CTCF binding site in Tc1 mouse liver. Upstream of the Shared H3K4me3 site at the *CSTB* gene, there is a Shared HNF4A and CEBPA binding event, while Alu-associated CEBPA and HNF4A sites are revealed in the Tc1 mouse. (B) Heatmap representation of repeats enriched in Tc1-specific events (red) or enriched in Shared (black) at H3K4me3 and transcription factor peak summits in liver, kidney, and testes. p values as calculated by chi-square test are presented in –log^10^ scale. Only repeat names that are significant in at least one data set are shown (p = 0.05).

At least 60% of Tc1-specific H3K4me3-associated regions enriched for repeats were identified in all somatic tissues profiled (Table S3). Of the most significantly enriched repeat types identified in the Tc1 mouse liver, the LTR elements LTR12C (10/11 on Tc1-HsChr21) and LTR12D (5/5 on Tc1-HsChr21) were constitutively activated across kidney, brain, muscle, and spleen Tc1 mouse tissues (Table S3A and Figure 4). Other significant liver-enriched repeats of the LINE, SVA, and SINE classes showed variable activation across tissues (Table S3).

Many of these repeat elements were primate and human lineage-specific and were transcriptionally silent in human liver and kidney (Figure 3D, Figure S3B). Indeed, based on analysis of the nucleotide substitution and mutation rates, these repetitive elements are significantly younger than those shared between human and Tc1 mice (Experimental Procedures, Figures 3A and 3B, righthand panels; Figure S3A), consistent with a mechanism wherein mice may lack the regulatory machinery needed to silence human-specific repetitive elements.

In the testes, the repeat enrichments were not significantly different between the Tc1-specific and Shared categories (p = 0.27) (Table S2B). In contrast to the human somatic tissues, many repetitive elements found in the Tc1 mouse testes are also enriched for H3K4me3 in human testes (e.g., 2/12 L1s, 1/5 SVAs, 7/8 AluYs, and 14/17 LTRs).

Between somatic and germline tissues, the most striking difference in transcription initiation among classes of repeat regions was observed for the SINE class (especially the AluY subfamily), which were significantly more often enriched for H3K4me3 in both human and Tc1 mouse testes (Figure 3C, middle panel; Figure S3, Table S3B). AluY is the youngest Alu subfamily member in human, originating less than 35 million years ago (Jurka et al., 2002), after the mammalian radiation. The many recently evolved SINE elements transcribed in both species’ testes shifted the repeat age distribution (Figure 3C, right panel); removal of this specific class of repeats results in age distributions similar to those found in somatic tissues (Figure S3A).

In contrast to mouse B1 SINE elements, which are often bound by Pol II at testes-specific promoters (Ichiyanagi et al., 2011), primate-specific Alu SINE elements are regulated by RNA polymerase III (Pol III) (Rogers, 1983). We performed Pol III ChIP-seq in Tc1 testes, and using multiply mapping reads, we observed 165 AluY-associated Pol III peaks that occurred within regions enriched for H3K4me3 in the same tissue (Table S4). While these results support the role of Pol III in regulating AluY elements in mouse testes, the low mappability of AluY repeats prevented us from assessing binding differences with other Tc1 mouse tissues. Overall, these results indicate that Pol III and its regulatory machinery can, at least in part, accurately interpret human AluYs in the heterologous mouse testes.

### Transcriptional Regulator Binding in the Tc1 Mouse

Given the widespread transcriptional activation of human-specific repeat elements in Tc1 mouse tissues, we asked whether they might also harbor latent transcription factor binding sites. Recent studies have demonstrated that up to a quarter of the OCT4 and NANOG stem-cell-specific transcription factor binding events in the human genome contain TEs (Kunarso et al., 2010); similarly, the binding evolution of neural restrictive silencing factor (NRSF) (Johnson et al., 2006) and the insulator protein CTCF binding can depend on repetitive elements (Bourque et al., 2008; Kunarso et al., 2010; Schmidt et al., 2012).

We investigated the genome-wide binding of CTCF (Schmidt et al., 2012) as well as the tissue-specific transcription factors CEBPA and HNF4A (Schmidt et al., 2010) in both human and Tc1 mouse liver tissue (Figure 4A and Figure S4A) and found a number of primate-specific repeats bound by these transcription factors (Figure S4B). For instance, an LTR12C repeat, comprising part of the long noncoding RNA *BC041449* that is located directly upstream of the amyotrophic lateral sclerosis gene *SOD1*, is occupied by CTCF in Tc1-mouse liver, but not in human. This CTCF binding event also shows trimethylation of H3K4 as well as Pol II occupancy, illustrating that repeat-driven latent regulatory potential could be biologically important (Figure S4C).

The set of TE families significantly enriched in a Tc1-specific manner for each transcription factor varied and was distinct from those revealed in our H3K4me3 ChIP-seq experiments (Figure 4B). For example, the Tc1-specific HNF4A binding events are particularly enriched for Alu SINE elements, an observation that is supported by recent analyses showing that these elements contain HNF4A binding motifs (Bolotin et al., 2011).

A greater fraction of the Tc1-specific CTCF binding sites are enriched for H3K4me3 (22/39) than are the Shared CTCF sites (90/358) (Table S5). In contrast, 40%–50% of CEBPA and HNF4A binding is associated with H3K4me3, regardless of whether the binding is Shared or Tc1-specific (Table S4). All CTCF-bound LTR elements are associated with H3K4 trimethylation (4/4) compared to a minority of CEBPA-bound (2/16) and HNF4A-bound (3/18) Alu elements. The absence of H3K4me3 at these transcription factor binding sites does not exclude the possibility that their binding was facilitated by the differential regulation of other epigenetic modifications, such as those found at enhancer elements. In sum, many different classes of repetitive elements in the human genome contain latent regulatory instructions for transcription factor binding, transcriptional activation, and polymerase occupancy that is revealed in vivo when placed in a heterologous mouse environment.

### Mouse-Nucleus-Mediated Changes in Human Chromosome 21 DNA Methylation Correspond to Regions of Unmasked Regulatory Potential

We sought to identify the mechanism underlying the activation of these normally latent human regulatory elements. Prior observations have noted that methylation of cytosines in CpG dinucleotides can cause transcriptional silencing of repetitive elements across eukaryotes (Zemach et al., 2010). Indeed, cytosine methylation is typically anticorrelated with H3K4me3 (Cedar and Bergman, 2009). Thus, we first asked if differences in the methylation of cytosines on HsChr21 associate with the transcriptional changes we observed.

**Figure 5:**
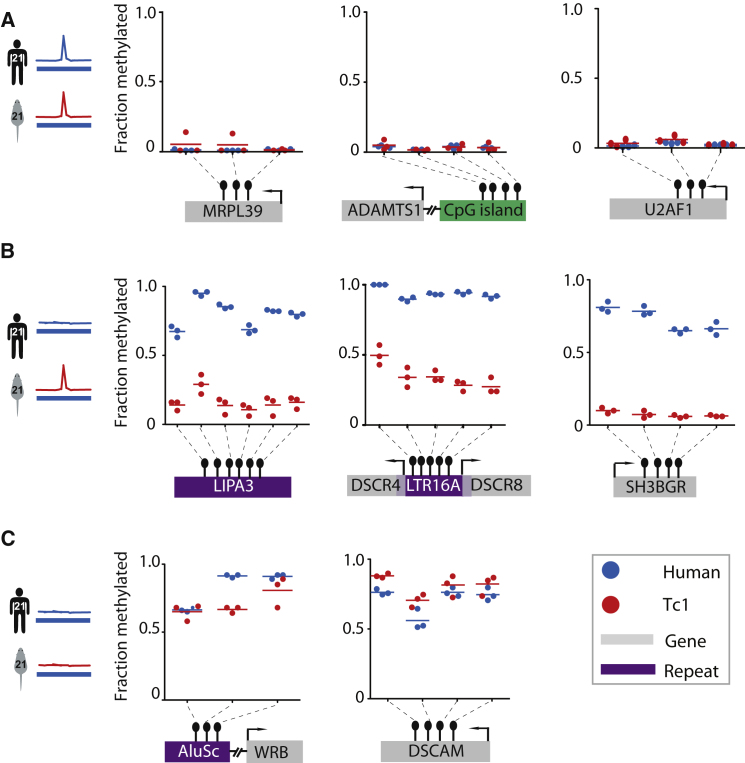
Tc1-Specific Sites of Transcription Initiation Are Depleted in DNA Methylation (A) Fraction of methylated DNA at H3K4me3-enriched sites Shared between HsChr21 (blue) and Tc1-HsChr21 (red) in liver. (B) Fraction of methylated DNA at Tc1-specific H3K4me3-enriched sites. (C) Fraction of methylated DNA at regions where there is no H3K4me3 enrichment in Human or Tc1 mouse. Interrogated CpG sites are shown within LTR and LINE repetitive elements (purple) or genes (gray). Each experiment was performed using three biological replicates.

To identify the CpG methylation state of representative HsChr21 regions, we performed bisulphite conversion of isolated genomic DNA from human and Tc1 mouse livers, followed by locus-specific pyrosequencing analysis. In regions of Shared H3K4me3 enrichment, CpG dinucleotides were consistently hypomethylated in both species (five regions with two to four CpG sites per region) (Experimental Procedures, Figure 5A, Tables S6A and S6B). Conversely, in regions lacking H3K4me3 enrichment, CpGs were uniformly methylated in both species (two regions with three CpG sites per region) (Figure 5C, Tables S6A and S6B). These results are consistent with the above-mentioned anticorrelation between DNA methylation and H3K4 trimethylation. Importantly, CpGs falling in Tc1-specific, H3K4me3-enriched regions in Tc1 mouse livers showed less CpG methylation compared to human livers. This trend was observed for multiple CpGs in both repeat-associated (LINE and LTR; n = 4), and nonrepetitive Tc1-specific H3K4me3 regions (n = 3) (Figure 5B, Tables S5A and S5B).

We extended these CpG methylation experiments to the entire HsChr21 by using Illumina Human Methylation 450k BeadArrays to assess CpG methylation in two tissues (liver and testes) obtained from human, Tc1 mice, and (as a hybridization control) wild-type mice (Experimental Procedures). We identified 3,174 human CpG probes on HsChr21 that did not crosshybridize with mouse DNA and used these to compare the methylation state of HsChr21 in mouse and human. Consistent with the locus-specific results above, we found that in the Tc1 mouse, CpG sites in Tc1-specific H3K4me3 regions in liver (n = 15) and testes (n = 12) were depleted of DNA methylation, compared to human (liver median fraction methylated CpG sites 0.6 [Hsa] versus 0.45 [Tc1], p < 0.05; testes median methylation 0.83 [Hsa] versus 0.39 [Tc1], p < 0.05) (Figure S5A and Table S6C). Interestingly, CpG sites within Shared H3K4me3 regions in liver (n = 43) and testes (n = 92) indicated an elevation of DNA methylation in the Tc1 mouse relative to human (liver median fraction methylated CpG sites was 0.06 [Hsa] versus 0.25 [Tc1], p < 0.005); testes median methylation was 0.11 [Hsa] versus 0.40 [Tc1], p < 0.005) (Figure S5A). Although this array-based analysis was limited by the small number of CpG probes in Tc1-specific H3K4me3 regions, these results suggest that DNA methylation plays a role in the differential epigenetic regulation of human DNA sequences in a heterologous mouse environment (Experimental Procedures, Figure S5A, Table S6A).

If DNA methylation changes in the Tc1 mouse were the mechanism that unmasked latent regulatory information in human repetitive sequences, then treating human cells with an agent that globally demethylates DNA should result in transcriptional upregulation of the same regions revealed by the Tc1 mouse. We treated cultured HepG2 liver cancer cells with 5-Aza-2′deoxycytidine-5′-triphosphate (5-Aza-dCTP) to globally demethylate cytosines (Dannenberg and Edenberg, 2006) and indeed observed that this subsequently resulted in trimethylation of H3K4 in the regions activated in Tc1 mouse tissues (n = 4, Figure S5B).

For instance, this treatment altered the DNA methylation of the LTR16A repeat element found at the *DSCR4/8* promoter, which is also upregulated in Tc1 mice (Figure 1B, Figure S5B). Consistent with the anticorrelation of DNA methylation and transcription, this promoter is hypomethylated in human placenta, where it is normally expressed, but hypermethylated in blood cells (Du et al., 2011). Importantly, no increase in H3K4me3 enrichment was observed for regions silenced in both human and mouse. However, regions not capable of further DNA demethylation (n = 4) also showed enrichment in H3K4me3, suggesting that the effect we observe could be due in part to indirect effects (Figure S5B). Indeed, a related cytosine analog designed to inhibit DNA methylation has been shown to additionally affect genomic organization (Komashko and Farnham, 2010).

In sum, DNA methylation changes mechanistically contribute to transcriptional activation of regions identified as latent regulatory elements by the Tc1 mouse, as global DNA demethylation in human cells can lead to transcriptional activation of these same regions.

### Changes in the Level of H3K9 Trimethylation Repressive Mark Occur at Human Regions that Are Specifically Activated in the Tc1 Mouse

**Figure 6:**
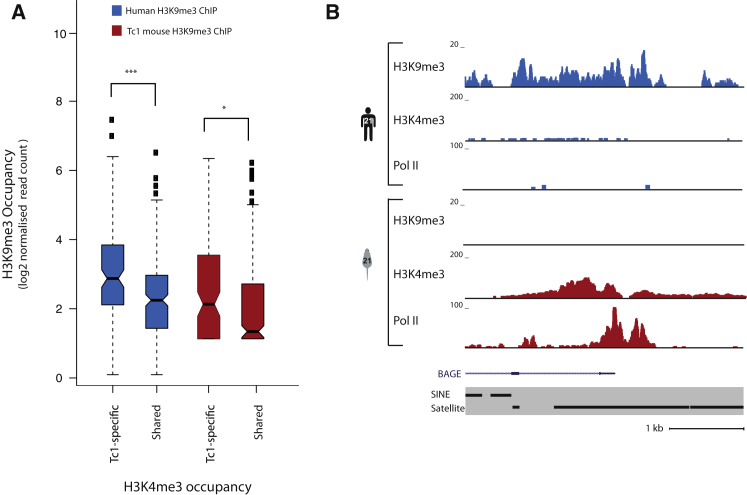
Changes in Transcription Status between Human and Tc1 Mouse Are Associated with Changes in the Repressive Histone Mark H3K9me3 (A) The genome-wide occupancy of H3K9me3 was determined in human liver (blue) and Tc1 mouse liver (red) by ChIP-seq and then compared with the transcriptional activation status of these regions. The vertical axis shows the log2 normalized read counts for H3K9me3 ChIP experiments averaged across three individuals from each species. (^∗^p ≤ 0.05, ^∗∗∗^p ≤ 0.0005, Wilcoxon-matched pairs test). (B) The occupancy of H3K9me3, H3K4me3, and Pol II is shown for the *BAGE* gene on HsChr21 located in human liver (blue) and Tc1 mouse liver (red). Human liver shows enrichment of the repressive histone mark H3K9me3 over the *BAGE* gene, which is missing in Tc1 mouse liver. Conversely, the mouse liver shows hallmarks of transcription (H3K4me3 and Pol II occupancies) that are absent from human.

Mechanistic studies in mouse embryonic stem cells have implicated the regulation of H3K9me3 in silencing proviral ERV elements through proteins such as KAP-1, the histone deacetylase (HDAC1), and ESET (Macfarlan et al., 2011; Matsui et al., 2010; Reichmann et al., 2012; Rowe et al., 2010). Trimethylation of H3K9 is associated with transcriptional repression, in contrast to trimethylation at H3K4, which is associated with transcriptional activation (Barski et al., 2007). We considered the possibility that H3K9me3 is important for silencing the active Tc1-specific H3K4me3-associated repeats in human tissue (Figure 6A).

We therefore profiled the genome-wide occupancy of H3K9me3 in human and Tc1 mouse liver using ChIP-seq (Experimental Procedures). We found that in human liver, Tc1-specific H3K4me3 regions showed elevated levels of H3K9me3 when compared to Shared H3K4me3 regions (Wilcoxon p = 7.9 × 10^−7^, Kolmogorov-Smirnov p = 1.5 × 10^−5^, distance = 0.28) (Figure 6A, blue boxplots). We also observed modest changes in the enrichment of H3K9me3 at Tc1-specific H3K4me3 regions relative to Shared regions in the Tc1 mouse, but there is less evidence for this enrichment (Wilcoxon p = 0.001, Kolmogorov-Smirnov p = 1.2 × 10^−1^, distance = 0.18) (Figure 6A, red boxplots). This result is consistent with the idea of trimethylation of H3K9 playing a role in sustaining transcriptional repression in human tissues; however, this mark is not anticorrelated with the trimethylation of H3K4 in our system (Figure 6A). It has previously been reported that there are site-specific effects of H3K9me3-mediated repression (Matsui et al., 2010; Karimi et al., 2011), and our data likely reflect this. Alternatively, the lost repression observed in the Tc1 mouse may be heterogeneous, such that the presence of apparently coexisting H3K9 and H3K4 trimethylation may actually indicate fluctuating activation and repression within a population of cells. This may also help explain why depletion of DNA methylation appears to transit to intermediate values, as opposed to complete demethylation.

At a few specific, repeat-containing loci, the occupancy of H3K9me3 in human appeared to be replaced by hallmarks of active transcription (Figure 6B). For instance, in human liver, the *BAGE* locus is strongly enriched for H3K9me3 across a genomic region containing a large REP522 satellite repetitive region; no evidence of occupancy by H3K4me3 or Pol II was apparent in human liver. Conversely, in Tc1 mouse liver, the *BAGE* locus appears to be actively transcribed and lacking the repressive histone mark H3K9me3. However, the majority of corresponding activated Tc1-specific regions in human liver show more modest changes.

We also attempted to perturb the H3K9 trimethylation-mediated silencing pathway identified in mouse embryonic stem cells, which has been shown to reactivate human-silent regions by inhibiting the activity of histone deacetylases (HDACs) using Trichostatin A in human HepG2 cells. However, this treatment was insufficient to activate latent sites, unlike treatment with 5-Aza-dCTP (Figure S6B).

## Discussion

The dynamic mechanisms underlying the ongoing evolution of the regulatory human genome are of profound interest, and the widespread involvement of repeat elements is only beginning to be understood (Bourque et al., 2008; Faulkner et al., 2009; Kunarso et al., 2010; Lowe et al., 2007; Lynch et al., 2011; Oliver and Greene, 2009; Schmidt et al., 2012; Xie et al., 2010). Although heterologous systems are routinely employed in cell culture and humanized mouse models (Devoy et al., 2012), it remains unknown how heterologous nuclear environments globally interact with species-specific repetitive elements in vivo. Using an entire human chromosome carried in an aneuploid mouse, we demonstrated that hundreds of normally silenced human loci have previously unsuspected regulatory potential. These loci show tissue-specific activation and are enriched for primate- and human-specific repeat elements utilized by transcription factors, insulator elements, and transcriptional machinery. We experimentally determined that the mechanism underlying this activation involves depletion of DNA methylation at LINE and LTR elements and a global decrease in repressive histone modifications in the Tc1 mouse.

These discoveries support a model wherein the regulatory impact of certain repeat elements in somatic tissues is diminished by DNA methylation, and possibly H3K9 trimethylation (De Fazio et al., 2011; Kondo and Issa, 2003; Levin and Moran, 2011; Matsui et al., 2010; Rebollo et al., 2011; Reuter et al., 2011). It follows that accurate regulation of recently evolved human-specific repeat elements depends on coevolved, species-specific silencing mechanisms. This can involve targeted repression by rapidly evolving zinc finger DNA-binding proteins (Huntley et al., 2006) or small RNA molecules that direct epigenetic machinery to specific genomic loci (Saito and Siomi, 2010). Indeed, the binding of tissue-specific transcription factors can establish regions of diminished DNA methylation (Stadler et al., 2011), and the subsequent, direct establishment of activating epigenetic modifications has been demonstrated using artificial unmethylated CpG clusters, which can recruit a CpG binding protein (Cfp1) and induce H3K4me3 de novo in the absence of Pol II and other transcription machinery (Thomson et al., 2010).

Our results also contribute to our understanding of germ cells as a highly active and transcriptionally exceptional tissue. The Tc1 mouse activates primate-lineage-specific AluY elements in the testes in a similar manner to what we observed in human testes, suggesting that the testes is uniquely suited to handling this class of elements. Thus, the testes-specific mechanisms of transcriptional activation seem to be more conserved than are the mechanisms of transcriptional silencing linked to changes in DNA methylation within somatic tissues.

Large-scale efforts using cells from a diverse range of primary human tissues are beginning to reveal the structure and function of the noncoding human genome (Consortium, 2011; Farnham, 2012; Dunham et al., 2012). Using a different strategy, we have exploited a single human chromosome transplanted into a heterologous regulatory environment to assess the transcriptional potential of most known human repeat families. The complete human genome is a hundred times larger, indicating that substantial latent regulatory potential remains to be discovered.

## Experimental Procedures

### Tissue Preparation

#### Mouse Material

The Tc1 mouse line was generated and maintained as previously described (O’Doherty et al., 2005). Tc1 mice were bred by crossing female Tc1 mice to male (129S8 × C57BL/6J) F1 mice and were housed in the Biological Resources Unit under UK Home Office licensing. Tissue was obtained from at least two independent males. Sibling Tc0 mice, which do not carry HsChr21, or C57BL/6J mice were used as a control. Fresh tissue was either flash frozen or crosslinked with 1% formaldehyde as previously described (Schmidt et al., 2009).

#### Human Material

Male and female human tissue samples were obtained from biopsied tissue collected at Addenbrooke’s Hospital, Cambridge, and were provided by the Biobank under human tissue license 08/H0308/117. Liver tissue was also obtained from the Liver Tissue Distribution Program (NIDDK contract number N01-DK-9-2310) at the University of Pittsburgh. For ChIP-seq, these samples were thawed in 1% formaldehyde and processed equivalently to the fresh crosslinked liver material used in this study.

### ChIP-seq

ChIP-seq was performed for H3K4me3, Pol II, Pol III, and H3K9me3 as described in Schmidt et al. (2009). The data for CEBPA and HNF4A (Schmidt et al., 2010) and CTCF (Schmidt et al., 2012) have been previously described and are deposited under accession numbers E-MTAB-722 and E-MTAB- 437, respectively.

### Sequence Alignment, Peak Calling, and Repeat Identification

ChIP-seq and input reads of 36–50 bp were aligned to the reference genome, human NCBI36 (hg18), the mouse genome (NCBIm37) (mm9) with the addition of HsChr21 (Tc1 genome), or a composite human + mouse genome, using the default parameters of the MAQ short DNA read alignment tool (http://maq.sourceforge.net/maq-man.shtml). Regions with a mapping quality score of 0 were removed, and uniquely mapping reads were used for subsequent enrichment analysis. Regions of H3K4me3 enrichment were determined using the Control-based ChIP-seq Analysis Tool (CCAT2.0) (Xu et al., 2010). Peaks were called at an FDR of 0.001 with a minimum 5-fold enrichment over input. As previously described, ChIP-enriched regions of CEBPA and HNF4A (Schmidt et al., 2010) and CTCF (Schmidt et al., 2012) were called using SWEMBL (http://www.ebi.ac.uk/∼swilder/SWEMBL/) with the parameters −R 0.005 −i −S.

Regions that mapped to known deleted or alpha satellite regions DNA on HsChr21 in the Tc1 mouse (O’Doherty et al., 2005) were excluded from analysis (precise regions were kindly provided by Dr. Susan Gribble). Peaks were overlapped with RepeatMasker (A.F.A. Smit, R. Hubley, and P. Green, RepeatMasker Open-3.0, 1996–2010, http://www.repeatmasker.org/) using custom Galaxy workflows (Blankenberg et al., 2010) (http://main.g2.bx.psu.edu/u/mdwilson/w/wilsonwardetalrepeatitemizationccat, http://main.g2.bx.psu.edu/u/mdwilson/w/wilsonwardetaltfrepeatitemizationswembl).

### Differential Binding Analysis

Enrichment analysis for all ChIP-seq experiments was performed as in Ross-Innes et al. (2012) using the DiffBind R/Bioconductor package (version 1.0) (R. Stark and G.D. Brown, DiffBind: differential binding analysis of ChIP-seq peak data, Bioconductor [2011] http://bioconductor.org/packages/release/bioc/html/DiffBind.html) for analysis of differential binding.

### RNA-Seq

#### Library Preparation and Sequencing

For the Tc1 mouse, mRNA-seq libraries were prepared from total RNA of three liver samples and sequenced on an Illumina Genome Analyzer II (50 bp single-end reads). For human liver, HiSeq 50 bp paired-end sequence reads from Illumina Human BodyMap 2.0 project were used. RefSeq transcripts were downloaded from NCBI (ftp://ftp.ncbi.nih.gov/genomes/H_sapiens/).

#### Alignment and Gene Expression Analysis

RNA-seq reads were mapped to RefSeq transcript sequences using Blat (Kent, 2002), and mapped reads were filtered as previously described (Pan et al., 2008). Gene expression levels were quantified in reads per kilobase of transcript per million mapped reads (RPKM) (Mortazavi et al., 2008).

### DNA Methylation Analysis

#### Locus-Specific DNA Methylation Assays

DNA was extracted from three Tc1, Tc0, and human liver tissues using the Easy-DNA Kit (Invitrogen) and bisulphite converted using the EZ DNA Methylation-Gold Kit (Zymo) according to manufacturers’ instructions. Regions of interest were amplified in a nested PCR reaction and the ratio of C:Ts determined using the PyroMark Q96 MD pyrosequencer (QIAGEN) and Pyro Q-CpG software (QIAGEN).

#### Illumina Infinium Human Methylation450K Bead Arrays

DNA was extracted and bisulphite treated from liver and testes flash frozen material from four human, Tc1, and Tc0 individuals as described above. Methylation profiling was performed using Illumina Infinium Human Methylation450K beadarrays according to the manufacturer’s standard protocol. Data were normalized using the Lumi BioConductor package (Du et al., 2008). Probes that showed significant detectable signal in Tc1 littermates that do not harbor Tc1-HsChr21 were excluded from further analysis. Arrays were run and analyzed by the Cambridge Genomics Service.

#### Abrogation of DNA Methylation in a Human Cell Line

HepG2 cells were treated with 1.5 μM 5-Aza-2′deoxycytidine-5′-triphosphate (5-Aza-dCTP) (Jena Bioscience) for 48 hr and retreated after 24 hr. Treated and untreated control cells were crosslinked with 1% formaldehyde 48 hr after initial treatment and H3K4me3 ChIP-qPCR performed as described above. DNA methylation levels were assayed to verify that DNA demethylation had occurred.

### Inhibition of HDAC Activity in a Human Cell Line

HepG2 cells were treated with DMSO or 500 nM Trichostatin A (TSA) (Sigma) for 24 hr prior to formaldehyde crosslinking followed by H3K4me3 ChIP-qPCR analysis as described above.

## Accession Numbers

The ArrayExpress accession number for the chromatin immunoprecipitation sequence reported in this paper is E-MTAB-1104. The ArrayExpress accession number for the human gene expression sequence reported in this paper is E-MTAB-513. The GEO accession number for the Tc1 gene expression sequence reported in this paper is GSE31454. The ArrayExpress accession number for the Illumina Infinium HumanMethylation450k beadarray data reported in this paper is E-MTAB-1304.

## Supplemental Information

Document S1. Figure S1, Figure S2, Figure S3, Figure S4, Figure S5, Figure S6, Table S1, Table S2, Table S3, Table S4, Table S5, Table S6, Supplemental Experimental Procedures, and Supplemental References

Document S2. GE Data; Repeat Class, Repeat Family, Repeat Name; Repeat Content HsChr21 H3K4
