# Supplementary material for: Latent Regulatory Potential of Human-Specific Repetitive Elements
Source: Mol Cell. 2013 Jan 24;49(2):262–72. doi: 10.1016/j.molcel.2012.11.013 (PMC3560060; doi:10.1016/j.molcel.2012.11.013)
Supplement: Document S1. Figure S1, Figure S2, Figure S3, Figure S4, Figure S5, Figure S6, Table S1, Table S2, Table S3, Table S4, Table S5, Table S6, Supplemental Experimental Procedures, and Supplemental References [file mmc1.pdf]

Molecular Cell, Volume *49*

Supplemental Information

Latent Regulatory Potential of Human-Specific Repetitive Elements

Michelle C. Ward, Michael D. Wilson, Nuno L. Barbosa-Morais, Dominic Schmidt, Rory Stark, Qun Pan, Petra C. Schwalie, Suraj Menon, Margus Lukk, Stephen Watt, David Thybert, Claudia Kutter, Kristina Kirschner, Paul Flicek, Benjamin J. Blencowe, and Duncan T. Odom

TABLE OF CONTENTS

SUPPLEMENTAL FIGURES

Figure S1: Gene expression in Human and Tc1 mouse, related to Fig. 1

Figure S2.A: Pipeline for classification of H3K4me3 regions, related to Fig. 2

Figure S2.B: ChIP-seq data replicate correlations, related to Fig. 2

Figure S2.C: Wildtype & Tc1 mouse H3K4me3 ChIP-seq data, related to Fig. 2

Figure S2.D: qPCR validation of H3K4me3 ChIP-seq data, related to Fig. 2

Figure S2.E: H3K4me3 ChIP-seq signal intensity distribution, related to Fig. 2

Figure S2.F: H3K4me3 ChIP-seq signal intensity values, related to Fig. 2

Figure S2.G: Association of H3K4me3 regions with Pol II, related to Fig. 2

Figure S2.H: H3K4me3 enrichment across Tc1 tissues, related to Fig. 2

Figure S3.A: Repeat enrichment at H3K4me3 peak summits, related to Fig. 3

Figure S3.B: Lineage of repeats at H3K4me3 peak summits, related to Fig. 3

Figure S4.A: Transcriptional regulator ChIP-seq data, related to Fig. 4

Figure S4.B: Repeat age and lineage in transcriptional regulator data, related to Fig. 4

Figure S4.C: Tc1-specific CTCF site upstream of *SOD1*, related to Fig. 4

Figure S5.A: Liver and testes DNA methylation array data, related to Fig. 5

Figure S5.B: Activation of latent sites after DNA de-methylation, related to Fig. 5

Figure S6.A: H3K9me3 ChIP-seq replicate correlations, related to Fig. 6

Figure S6.B: Lack of activation of latent sites after HDAC inhibition, related to Fig. 6

SUPPLEMENTAL TABLES

Table S1.A: Library read and peak summary, related to Fig. 2

Table S1.B: Alignment of wildtype and Tc1 mouse ChIP-seq data, related to Fig. 2

Table S1.C: Wildtype and Tc1 mouse ChIP-seq enriched regions, related to Fig. 2

Table S1.D: ChIP-seq data alignment to human-mouse genome, related to Fig. 2

Table S1.E: qPCR validation regions and primers, related to Fig. 2

Table S2.A: Fraction of ChIP-seq peaks overlapping repeats, related to Fig. 3

Table S2.B: Enrichment of repeat elements in ChIP-seq data, related to Fig. 3

Table S2.C: Repeat content of human chromosome 21, related to Fig. 3

Table S3: Inter-tissue H3K4me3 comparison, related to Fig. 3

Table S4: Pol III associates with activated AluY repeats in Tc1 testes, related to Fig. 3

Table S5: Transcriptional regulator site association with H3K4me3, related to Fig. 4

Table S6.A: CpG methylation values at interrogated H3K4me3 sites, related to Fig. 5

Table S6.B: Regions interrogated for DNA methylation analysis, related to Fig. 5

Table S6.C: CpGs interrogated on human DNA methylation array, related to Fig. 5

SUPPLEMENTAL EXPERIMENTAL PROCEDURES

SUPPLEMENTAL REFERENCES

Molecular Cell, Volume *49*

Supplemental Information

Latent Regulatory Potential of Human-Specific Repetitive Elements

Michelle C. Ward, Michael D. Wilson, Nuno L. Barbosa-Morais, Dominic Schmidt, Rory Stark, Qun Pan, Petra C. Schwalie, Suraj Menon, Margus Lukk, Stephen Watt, David Thybert, Claudia Kutter, Kristina Kirschner, Paul Flicek, Benjamin J. Blencowe, and Duncan T. Odom

TABLE OF CONTENTS

SUPPLEMENTAL FIGURES

Figure S1: Gene expression in Human and Tc1 mouse, related to Fig. 1

Figure S2.A: Pipeline for classification of H3K4me3 regions, related to Fig. 2

Figure S2.B: ChIP-seq data replicate correlations, related to Fig. 2

Figure S2.C: Wildtype & Tc1 mouse H3K4me3 ChIP-seq data, related to Fig. 2

Figure S2.D: qPCR validation of H3K4me3 ChIP-seq data, related to Fig. 2

Figure S2.E: H3K4me3 ChIP-seq signal intensity distribution, related to Fig. 2

Figure S2.F: H3K4me3 ChIP-seq signal intensity values, related to Fig. 2

Figure S2.G: Association of H3K4me3 regions with Pol II, related to Fig. 2

Figure S2.H: H3K4me3 enrichment across Tc1 tissues, related to Fig. 2

Figure S3.A: Repeat enrichment at H3K4me3 peak summits, related to Fig. 3

Figure S3.B: Lineage of repeats at H3K4me3 peak summits, related to Fig. 3

Figure S4.A: Transcriptional regulator ChIP-seq data, related to Fig. 4

Figure S4.B: Repeat age and lineage in transcriptional regulator data, related to Fig. 4

Figure S4.C: Tc1-specific CTCF site upstream of *SOD1*, related to Fig. 4

Figure S5.A: Liver and testes DNA methylation array data, related to Fig. 5

Figure S5.B: Activation of latent sites after DNA de-methylation, related to Fig. 5

Figure S6.A: H3K9me3 ChIP-seq replicate correlations, related to Fig. 6

Figure S6.B: Lack of activation of latent sites after HDAC inhibition, related to Fig. 6

SUPPLEMENTAL TABLES

Table S1.A: Library read and peak summary, related to Fig. 2
Table S1.B: Alignment of wildtype and Tc1 mouse ChIP-seq data, related to Fig. 2
Table S1.C: Wildtype and Tc1 mouse ChIP-seq enriched regions, related to Fig. 2
Table S1.D: ChIP-seq data alignment to human-mouse genome, related to Fig. 2
Table S1.E: qPCR validation regions and primers, related to Fig. 2
Table S2.A: Fraction of ChIP-seq peaks overlapping repeats, related to Fig. 3
Table S2.B: Enrichment of repeat elements in ChIP-seq data, related to Fig. 3
Table S2.C: Repeat content of human chromosome 21, related to Fig. 3
Table S3: Inter-tissue H3K4me3 comparison, related to Fig. 3
Table S4: Pol III associates with activated AluY repeats in Tc1 testes, related to Fig. 3
Table S5: Transcriptional regulator site association with H3K4me3, related to Fig. 4
Table S6.A: CpG methylation values at interrogated H3K4me3 sites, related to Fig. 5
Table S6.B: Regions interrogated for DNA methylation analysis, related to Fig. 5
Table S6.C: CpGs interrogated on human DNA methylation array, related to Fig. 5

EXPERIMENTAL PROCEDURES

SUPPLEMENTAL REFERENCES

SUPPLEMENTAL FIGURES

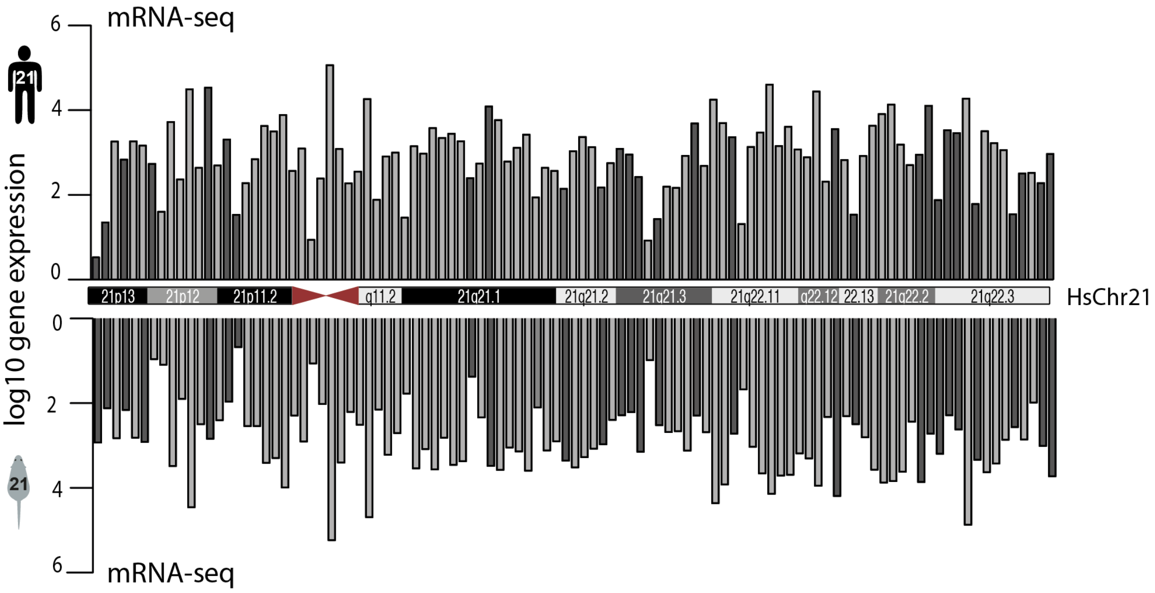

Figure S1: Gene expression patterns are similar between HsChr21 and Tc1-HsChr21, related to Figure 1. Human and Tc1 mouse mRNA-seq data of the 103 genes on HsChr21 that have orthologues in mouse and for which expression was observed are shown as normalised log expression values in the order in which they appear along HsChr21. The 28 genes differentially expressed as determined by a fold change of ≥ 4 between Human and Tc1 are shown in dark grey.

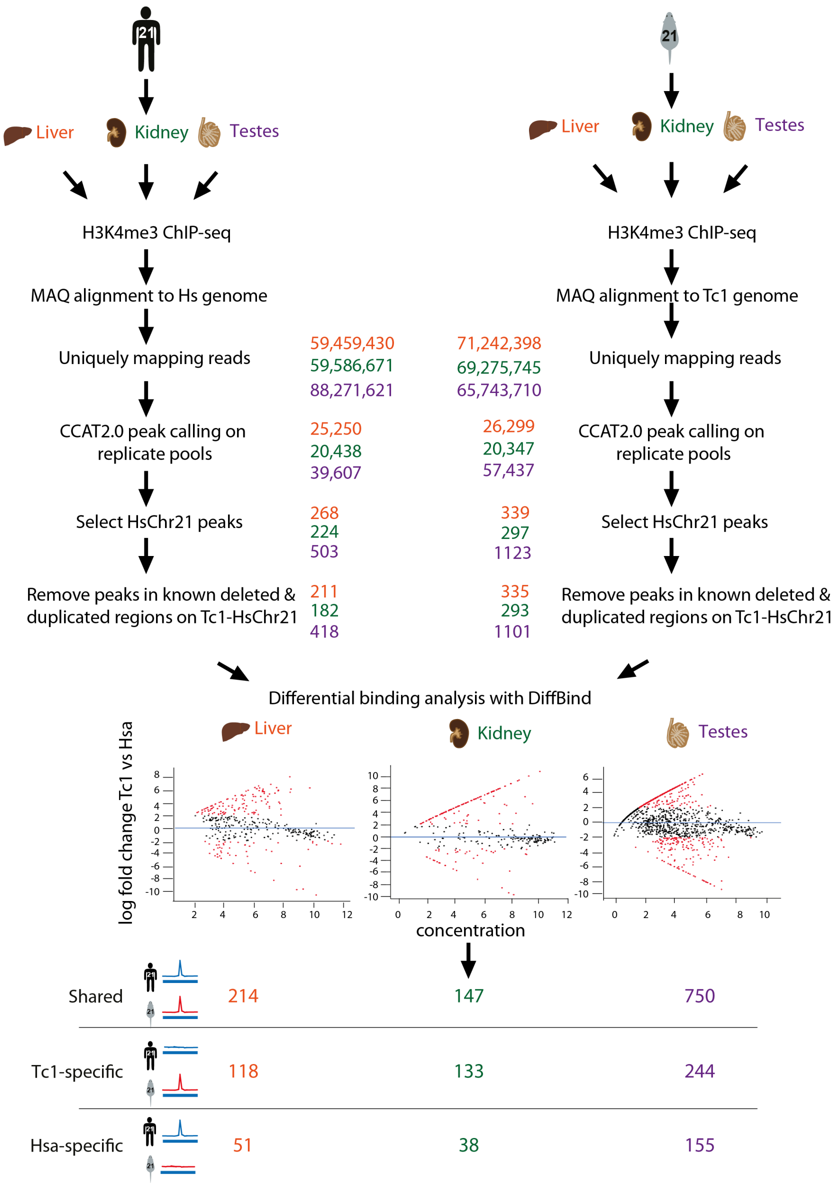

Figure S2.A: Pipeline for classification of regions of transcription initiation in human and Tc1 mouse, related to Figure 2. At least two biological replicates from human and Tc1 liver (orange), kidney (green) and testes (purple) tissue were used for H3K4me3 ChIP-seq analysis. Sequenced reads were aligned to the human (hg18) or Tc1 (mm9 plus HsChr21 (hg18)) genomes using MAQ. Uniquely mapping reads from a union of pooled datasets were used to identify regions of ChIP enrichment with the CCAT2.0 peakcaller (FDR<0.001 and >5-fold enrichment of ChIP over input). The DiffBind Bioconductor package was used to identify regions of differential enrichment between the two species (FDR<0.1 and >4-fold difference in normalised reads). All data is presented from the Tc1 perspective. H3K4me3 events that are not significantly differentially bound (black; Shared) and significantly differentially bound in Tc1 and Human (red; Tc1-specific y>2 and Human-specific y<-2) are shown in the MA plots.

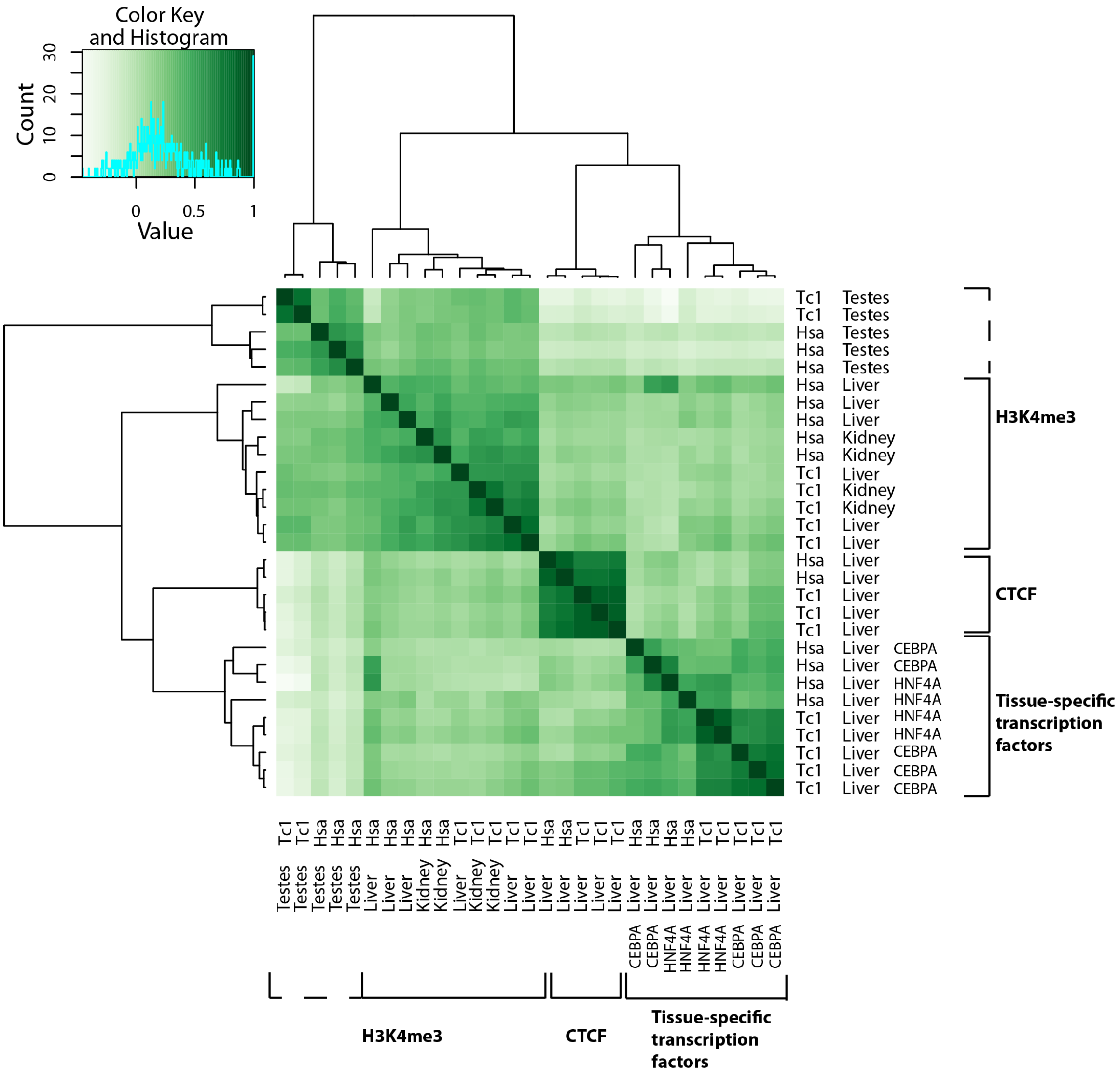

Figure S2.B: ChIP-seq experiments cluster factors as replicates, related to Figure 2. RPKM values of replicate ChIP-seq samples, relative to input DNA, were calculated to determine regions of enrichment for H3K4me3 in liver, kidney and testes, CTCF and the transcription factors CEBPA and HNF4A in liver using DiffBind Bioconductor package. Replicates cluster primarily by factor except for CEBPA and HNF4A that cluster first on species. H3K4me3 testes data forms a cluster distinct from H3K4me3 data in somatic tissue. Counts are reflective of Pearson correlation values between sample pairs and the heatmap generated using unsupervised hierarchical clustering.

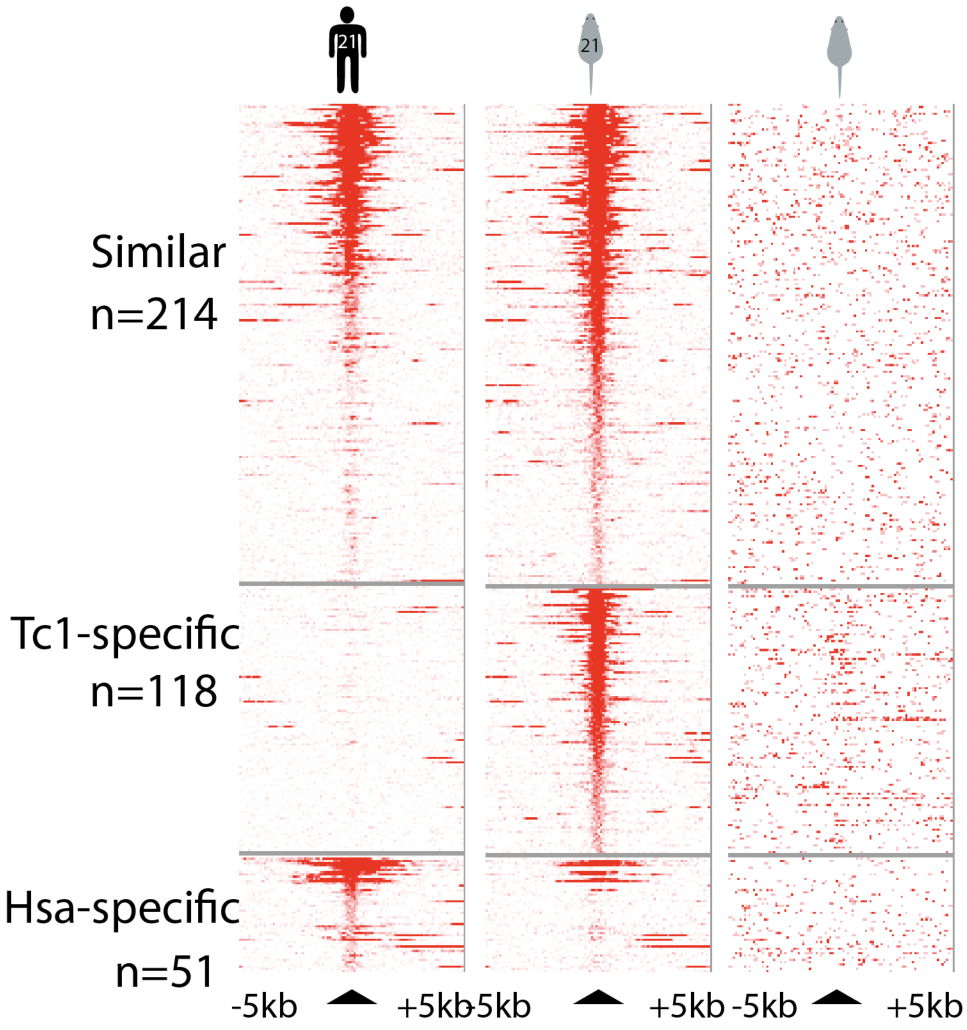

Figure S2.C: Wild type Tc0 mice reads do not cross-map to HsChr21, related to Figure 2. Heatmap representation of H3K4me3 ChIP-seq experiments in human, Tc1 and wild type Tc0 littermate liver. Human ChIP-seq data was aligned to the hg18 human assembly and the Tc1 and Tc0 control ChIP-seq experiments were aligned to the Tc1 genome (mm9 plus HsChr21 (hg18)).

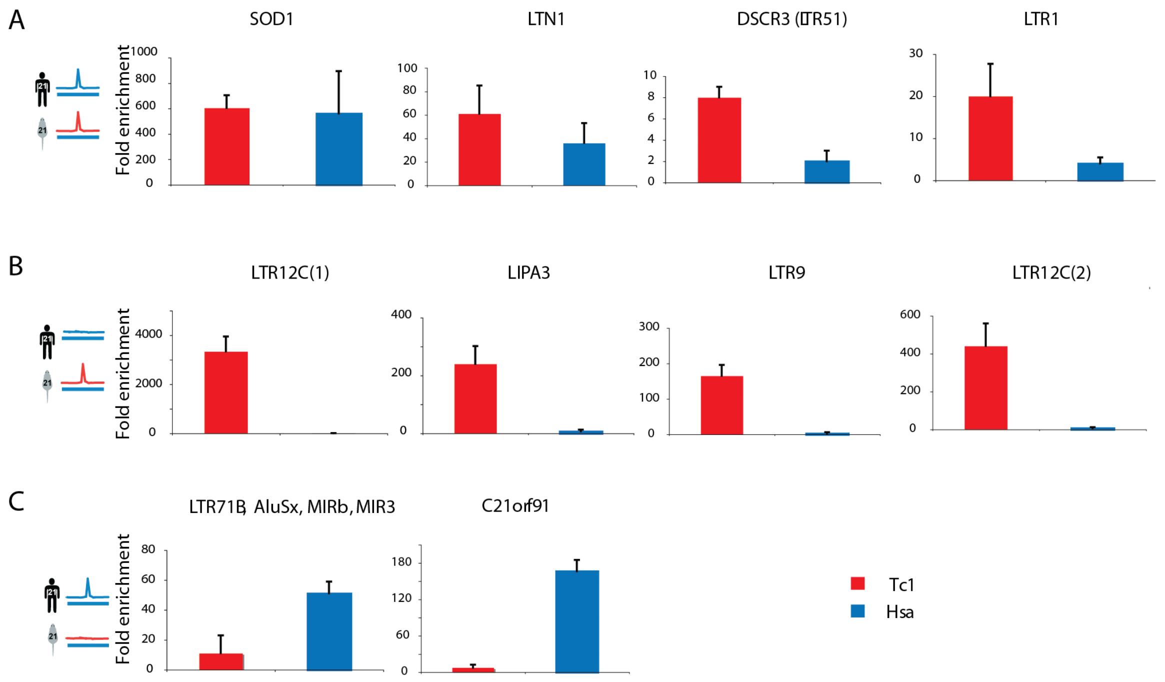

Figure S2.D: qPCR validates ChIP-seq H3K4me3 regions, related to Figure 2. Primers were designed around regions of H3K4me3 enrichment and two negative control regions. Results were normalised relative to the negative control regions and input DNA. A) H3K4me3 sites present in Human and Tc1 as defined as Shared by ChIP-seq analysis for human (blue) and Tc1 (red). B) H3K4me3 sites specific to Tc1. C) H3K4me3 sites specific to Human. The *C21orf91* gene is known to be deleted on Tc1-HsChr21. Error bars represent standard error of the mean. Chromosome coordinates (hg18) for H3K4me3 regions used and primer sequences are shown in Table S1.E.

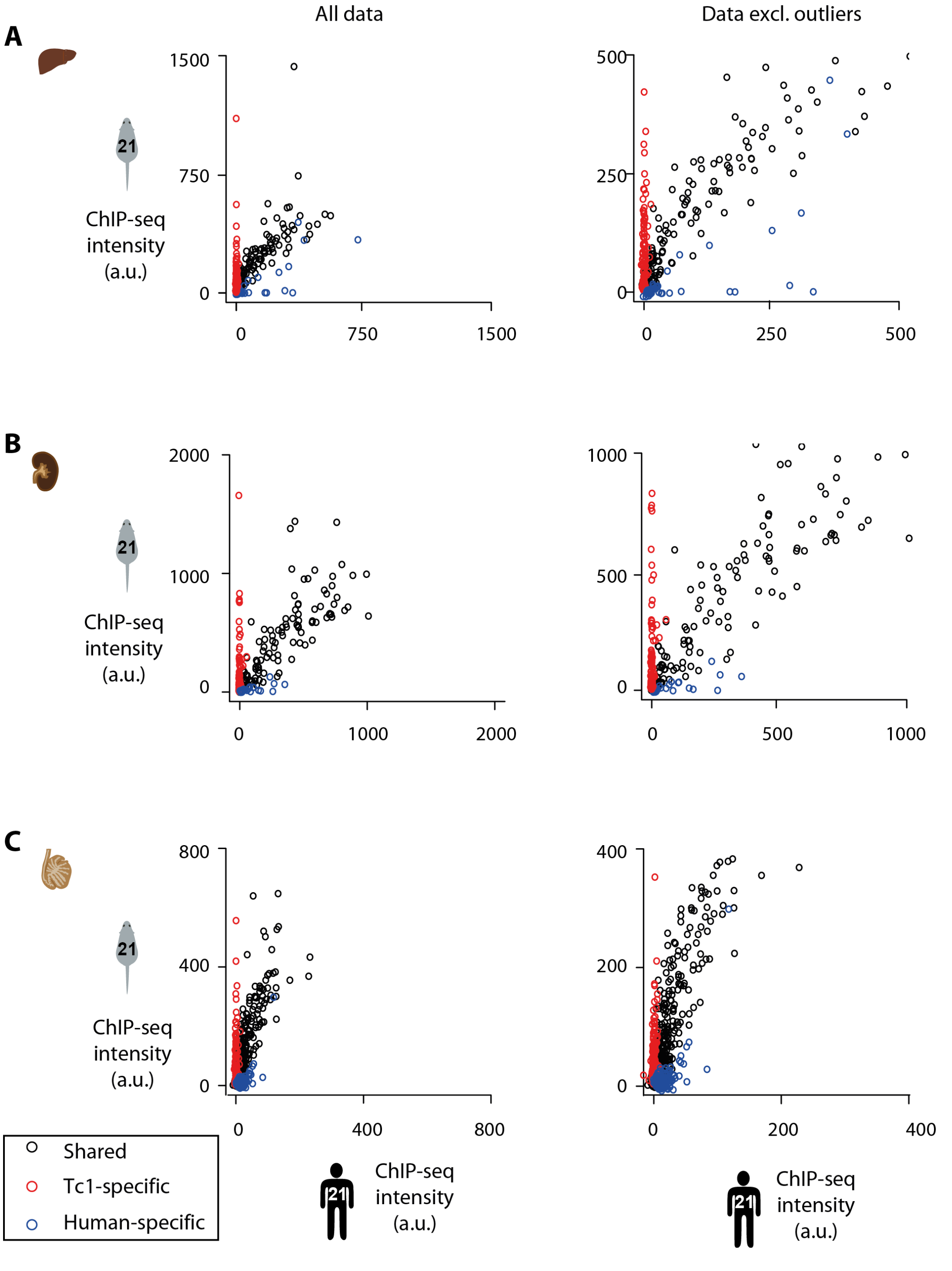

Figure S2.E: Tc1-specific H3K4me3 sites have more intense ChIP-seq signal in Tc1 mouse tissue compared to human, related to Figure 2. Average ChIP-seq intensity across 500 bp around the H3K4me3 peak summit normalised to input DNA in A) liver, B) kidney and C) testes. ChIP-seq intensity is represented as arbitrary units. Data excluding outliers is a zoomed in picture of the graph on the left.

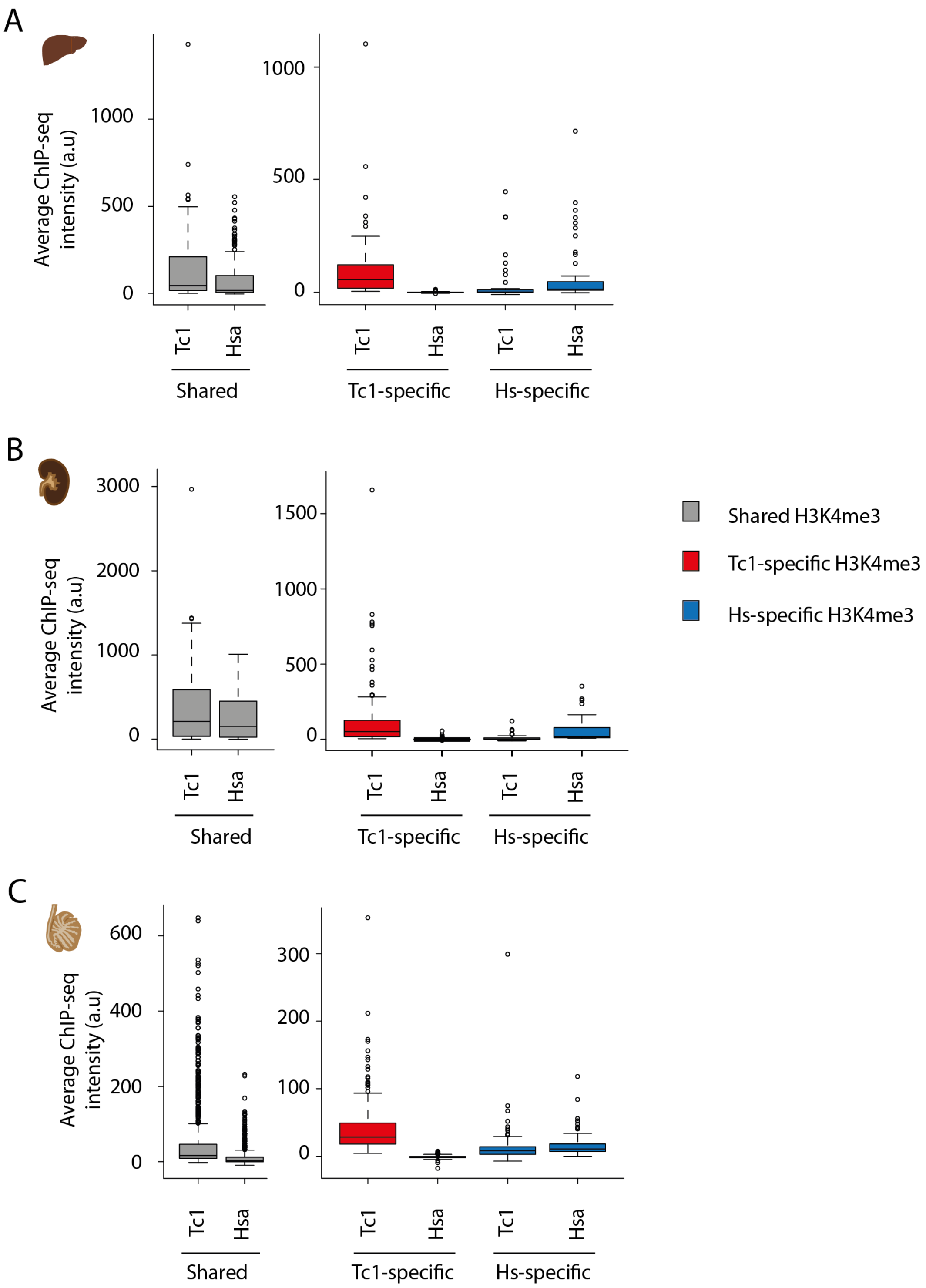

Figure S2.F: Tc1-specific H3K4me3 sites have more intense ChIP-seq signal in Tc1 mouse tissue compared to human, related to Figure 2. Average ChIP-seq intensity across 500 bp around the H3K4me3 peak summit normalised to input DNA in A) liver, B) kidney and C) testes. Human-specific sites are generally lower in intensity than Tc1-specific sites.

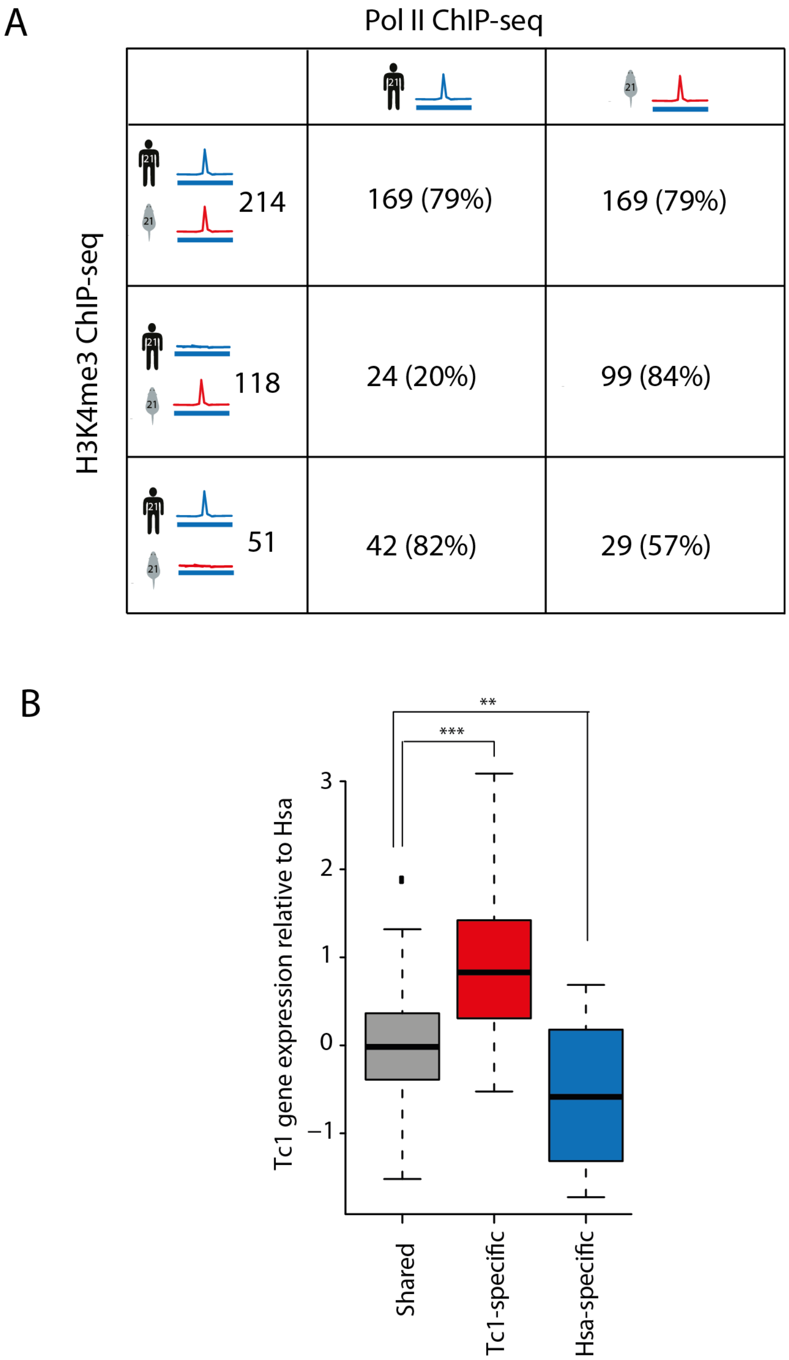

Figure S2.G: Many Tc1-specific H3K4me3 regions show Pol II occupancy, related to Figure 2. A) Numbers of Shared, Tc1-specific and Human-specific H3K4me3 enriched regions that overlap with human and Tc1 Pol II in human liver. The majority of Shared H3K4me3 sites show Pol II occupancy in both human and Tc1 mouse while Tc1-specific sites are preferentially associated with Pol II in Tc1 mouse. B) Log ratio of Tc1 gene expression relative to human gene expression of genes nearest Shared, Tc1-specific and Human-specific H3K4me3 regions. Shared n=86, Tc1-specific n=17, Human-specific n=11. (***p-value≤0.0005 **p-value≤0.005, one-sided Mann-Whitney U test).

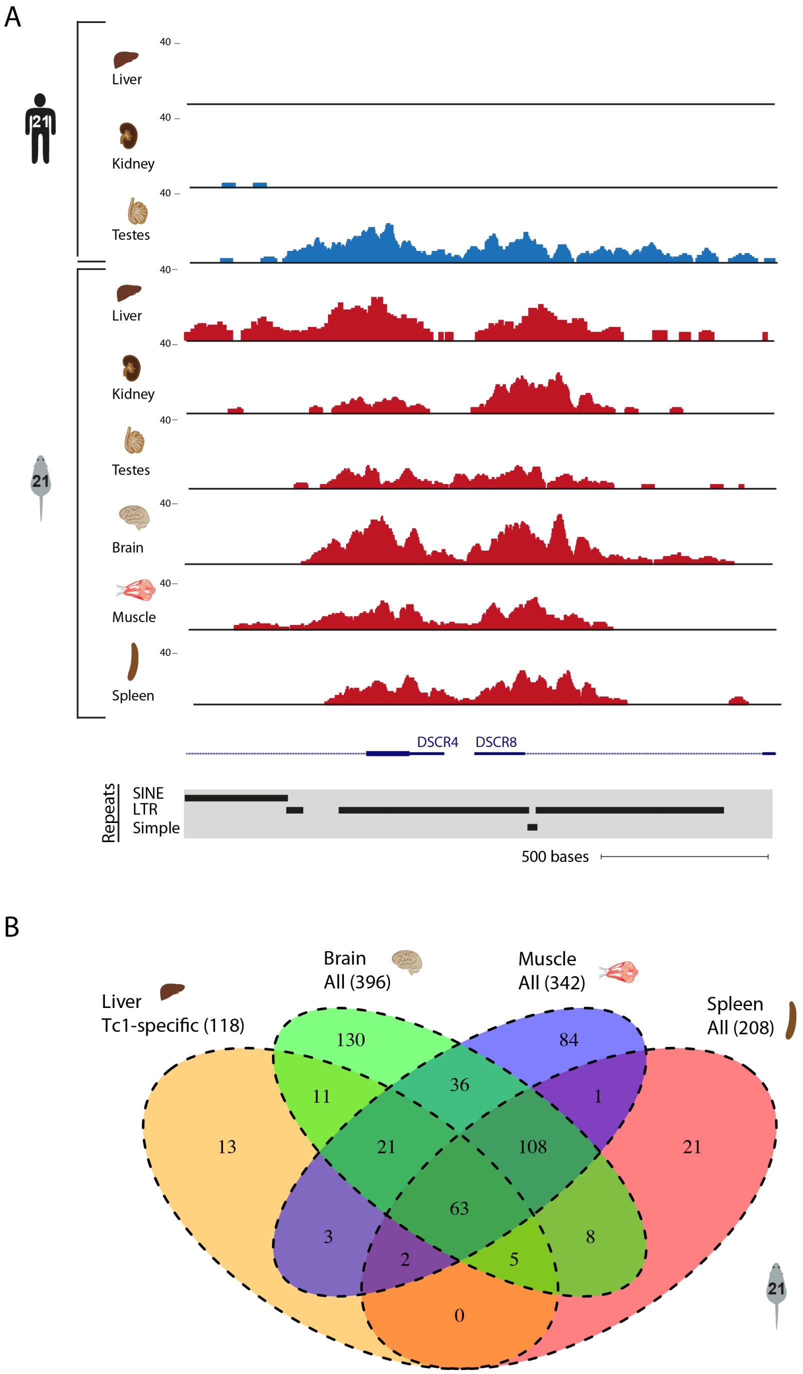

Figure S2.H: Many liver Tc1-specific H3K4me3 regions are found across Tc1 somatic tissues, related to Figure 2. A) H3K4me3 data from human liver, kidney and testes and Tc1 liver, kidney, testes, brain, spleen and muscle at the LTR driven bi-directional promoter at *DSCR4/8*. B) Liver Tc1-specific H3K4me3 regions (118/384 total between human and Tc1) overlapped with all H3K4me3 regions identified in Tc1 brain (396), muscle (342) and spleen (208). Data representative of at least two independent experiments per tissue.

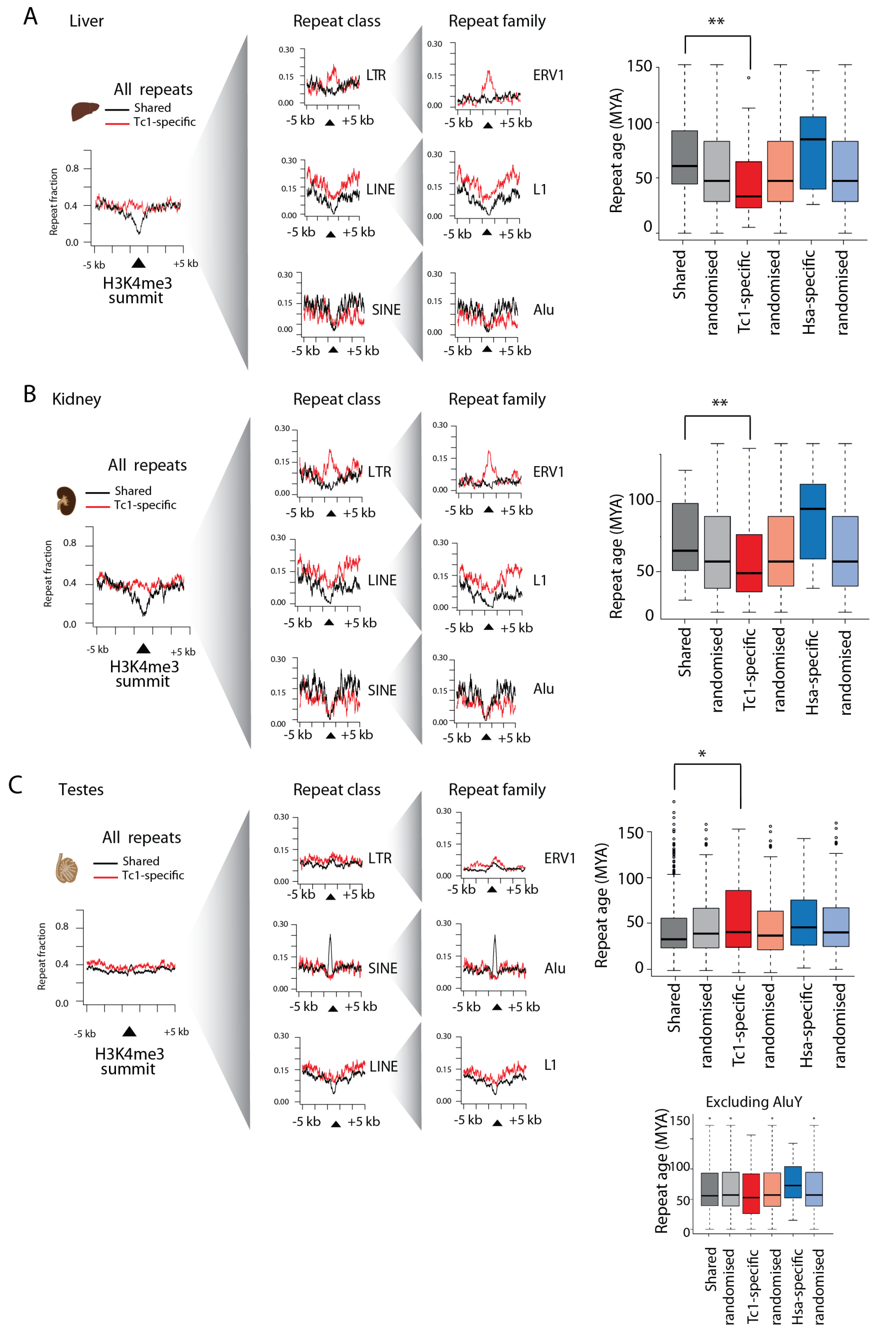

Figure S3.A: Particular repeat families are enriched in Tc1-specific H3K4me3 regions, related to Figure 3. A) Total fraction of repeats at H3K4me3 peak summits in Tc1-specific and Shared H3K4me3 binding events in liver. Age of the repeats at the H3K4me3 peak summits in Shared, Tc1-specific, Human-specific H3K4me3 sites as well as randomly generated peak sets. B) Fraction of repeats together with the repeat age in kidney H3K4me3 binding events. C) Fraction of repeats together with the repeat age in testes H3K4me3 binding events. The age of repeats excluding the AluY repeat types is shown in the inset where the age profile now resembles that of somatic tissue. The significance of the difference in ages of repeats between Shared and Tc1-specific H3K4me3 events was calculated by the Wilcoxon rank sum test. * p≤0.05, ** p≤0.005.

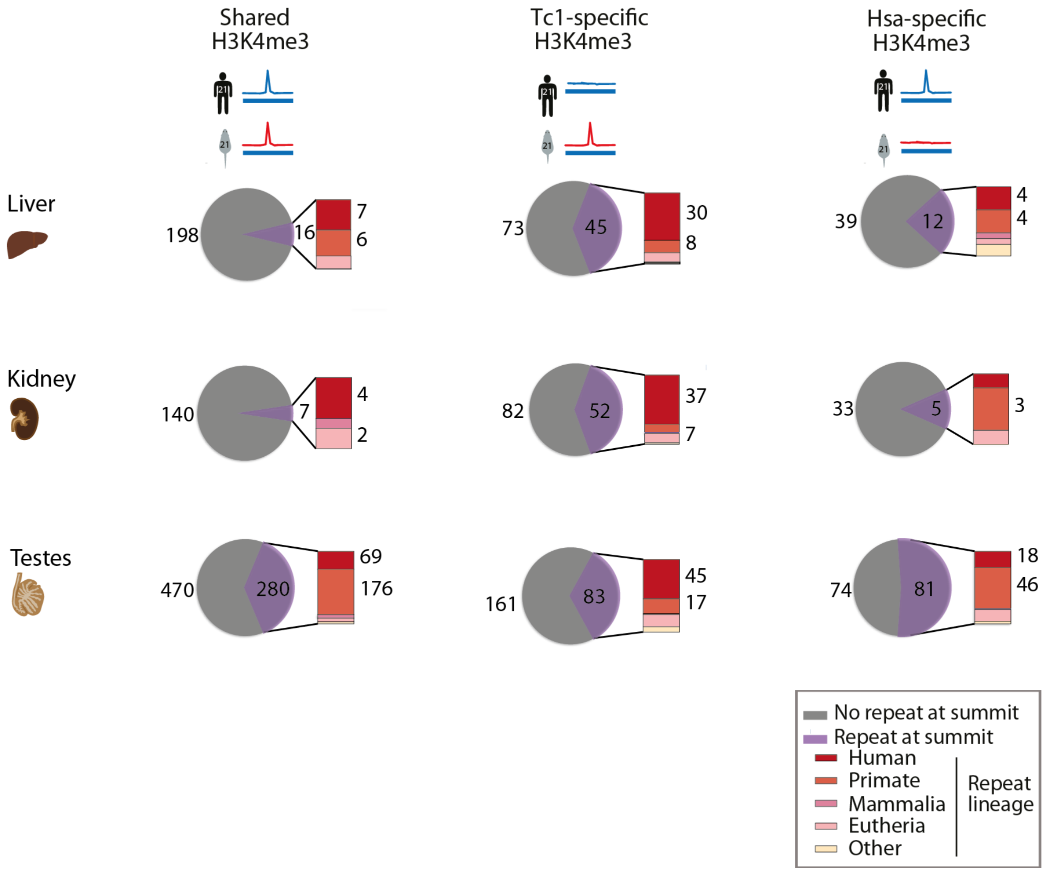

Figure S3.B: Tc1-specific sites of transcription initiation are enriched for human-specific repeats, related to Figure 3. Fraction of H3K4me3 enriched regions containing a repeat at the peak summit is shown in purple where the distribution of the lineages of the repeats are shown in the bar charts. Human-specific sites have low numbers of repeats in somatic tissue but a high number in testes.

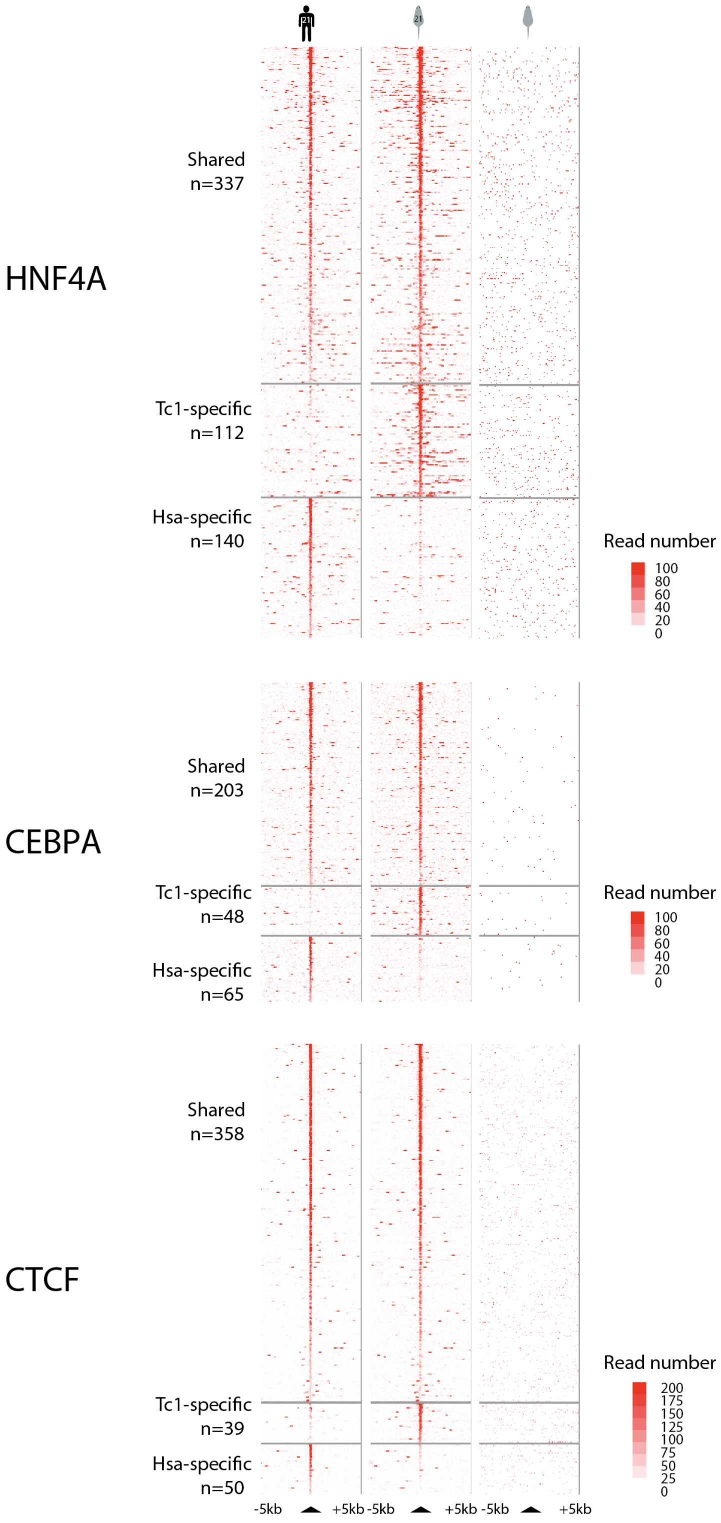

Figure S4.A: Chromosome-wide transcriptional regulator binding data reveals Tc1-specific sites, related to Figure 4. All HNF4A, CEBPA and CTCF binding sites in Human, Tc1 and Tc0 liver samples. Binding regions are centred on peak summits within a 10 kb window. Data is representative of at least two independent experiments.

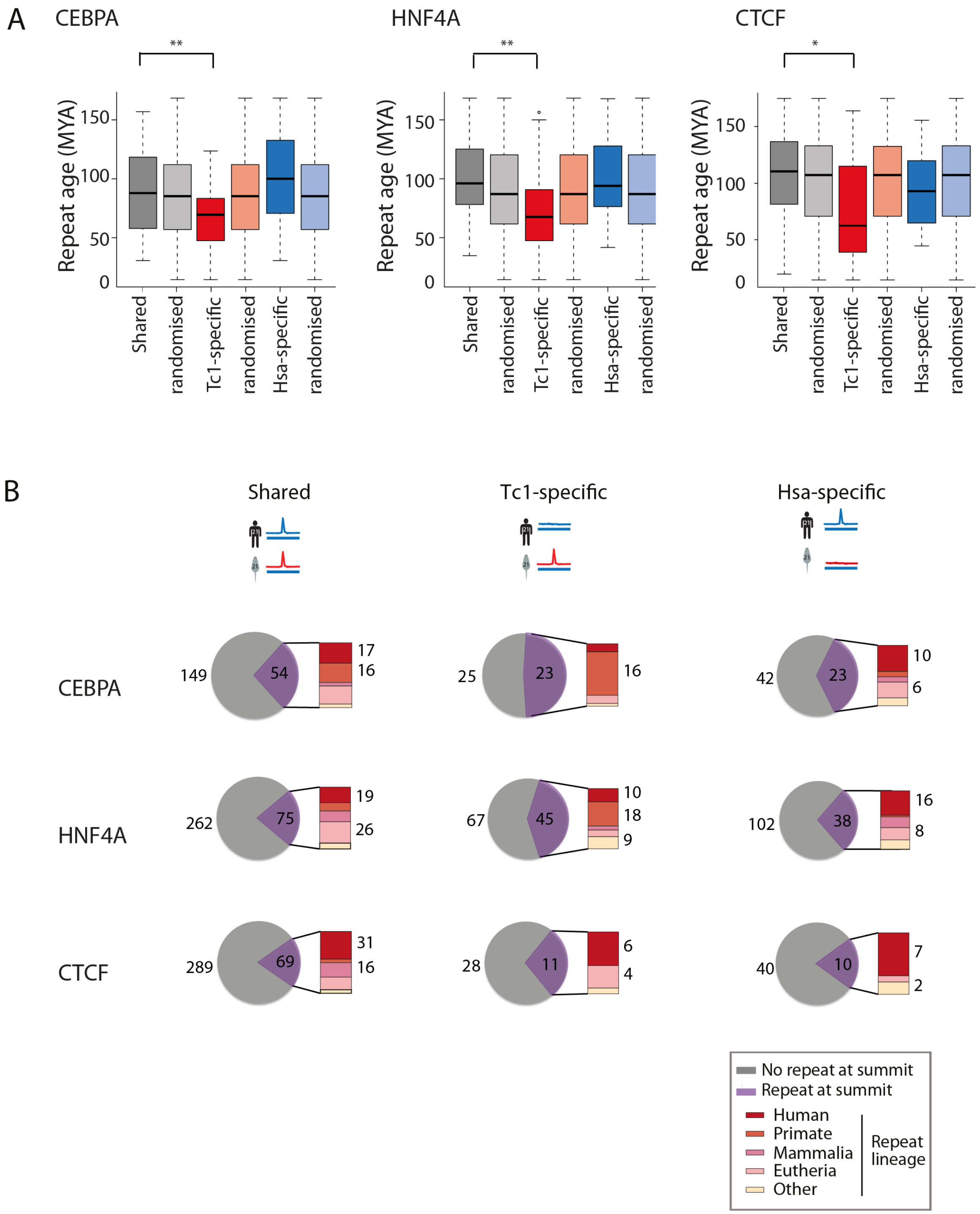

Figure S4.B: Tc1-specific transcriptional regulator binding events enrich for recent lineage-specific repetitive elements, related to Figure 4. A) Age of repeats in Shared and Tc1-specific CEBPA, HNF4A and CTCF binding events. B) Number and lineage of repeat elements at CEBPA, HNF4A and CTCF peak summits.
(* p≤0.05 and ** p≤0.005, Wilcoxon rank sum test).

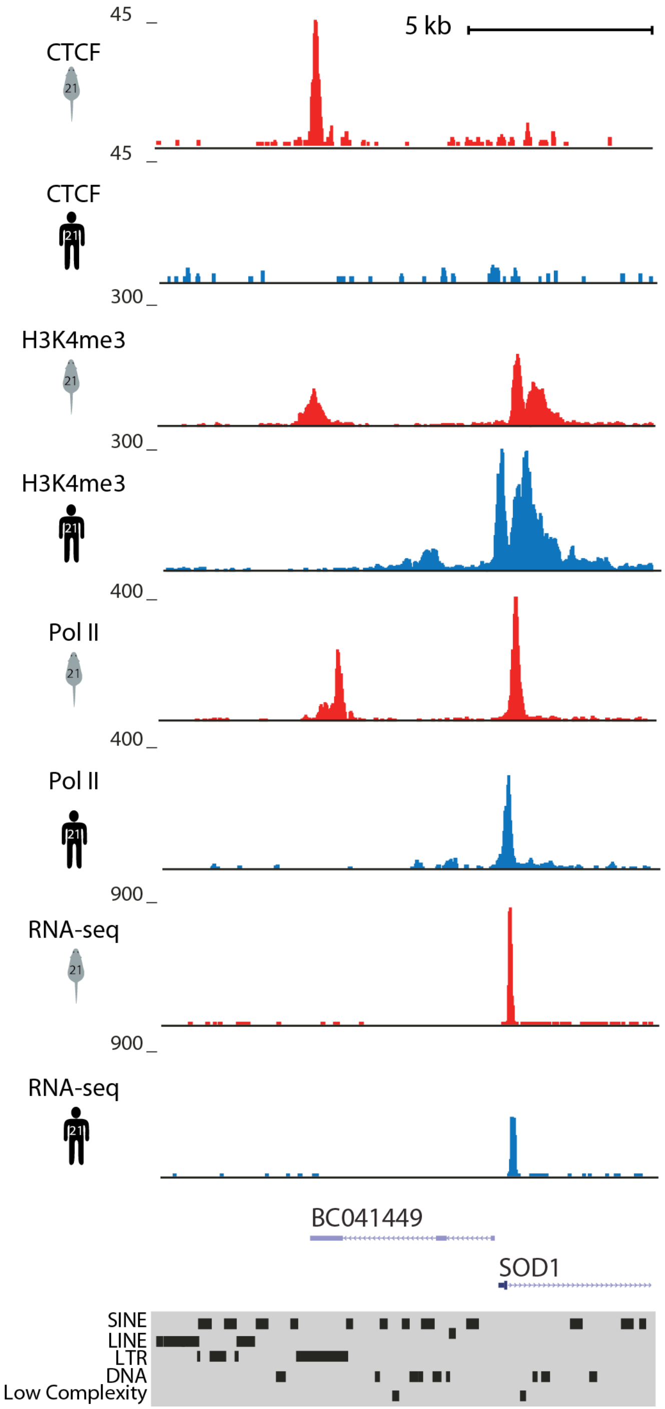

Figure S4.C: Tc1-specific CTCF site upstream of *SOD1* also shows H3K4 trimethylation and Pol II, related to Figure 4. CTCF, H3K4me3, Pol II and RNA-seq data from human (blue) and Tc1 (red) is shown at the ncRNA upstream of the *SOD1* transcription start site.

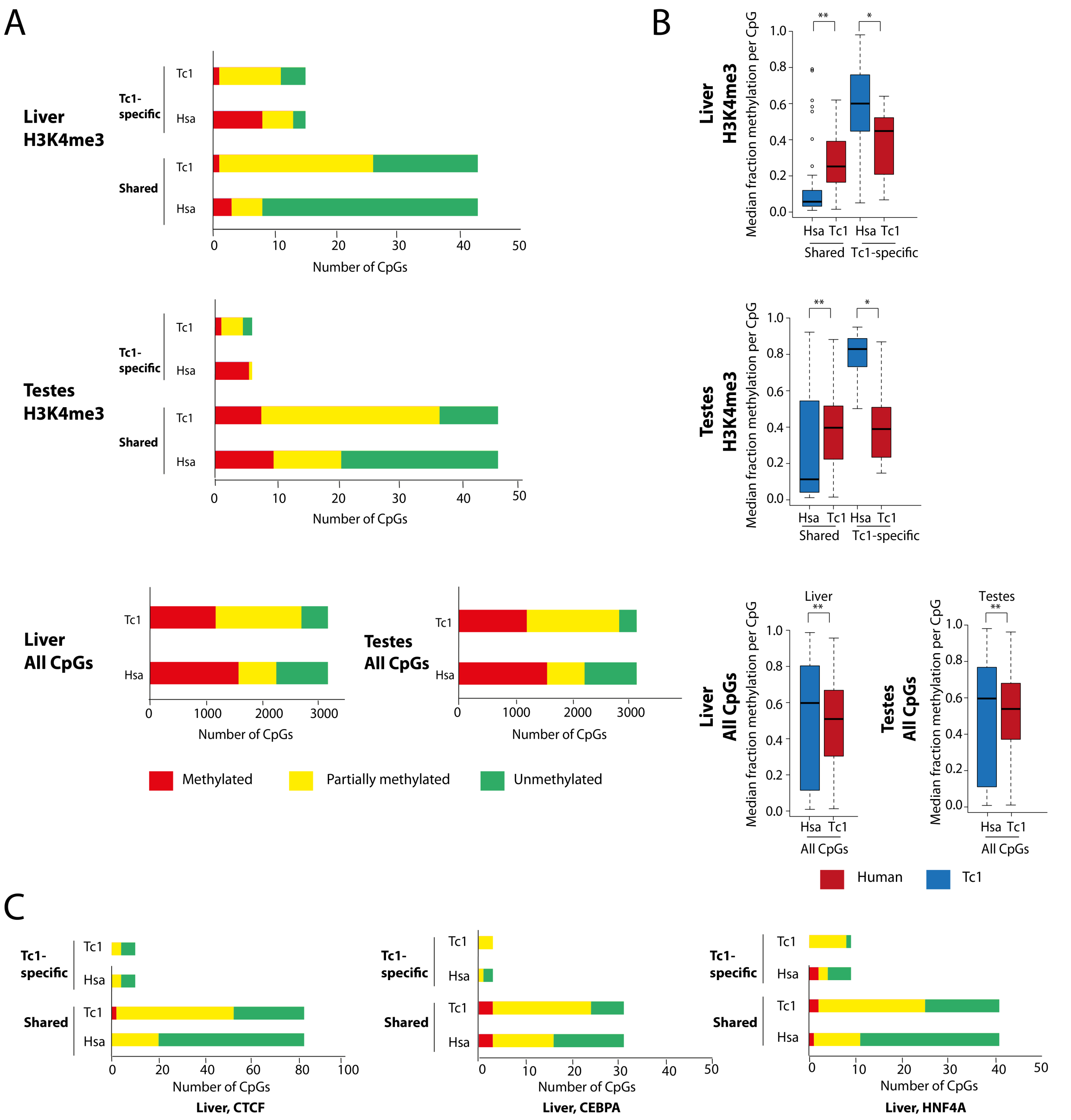

Figure S5.A: Tc1-specific H3K4me3 regions are more often associated with methylated DNA in human than in Tc1 mice, related to Figure 5. A) CpG methylation levels in Tc1 and human liver and testes DNA were determined by the Illumina Infinium Human Methylation450k beadarray. CpGs with significantly detectable signal in Tc0 mice were excluded from the analysis leaving 3174 CpG sites to interrogate across the chromosome. DNA methylation was classified into Methylated (>80% DNA methylation), Partially methylated (20-80% DNA methylation) and Unmethylated (<20% DNA methylation). Total numbers of interrogated CpG sites located within 100 bp of the summit of Tc1-specific and Shared H3K4me3 regions within each methylation category is shown. B) Median CpG methylation levels in human and Tc1 within Shared and Tc1-specific H3K4me3 regions. C) Classification of DNA methylation levels in CTCF, CEBPA and HNF4A Tc1-specific and Shared sites (* p≤0.05 and ** p≤0.005, Wilcoxon matched pairs test).

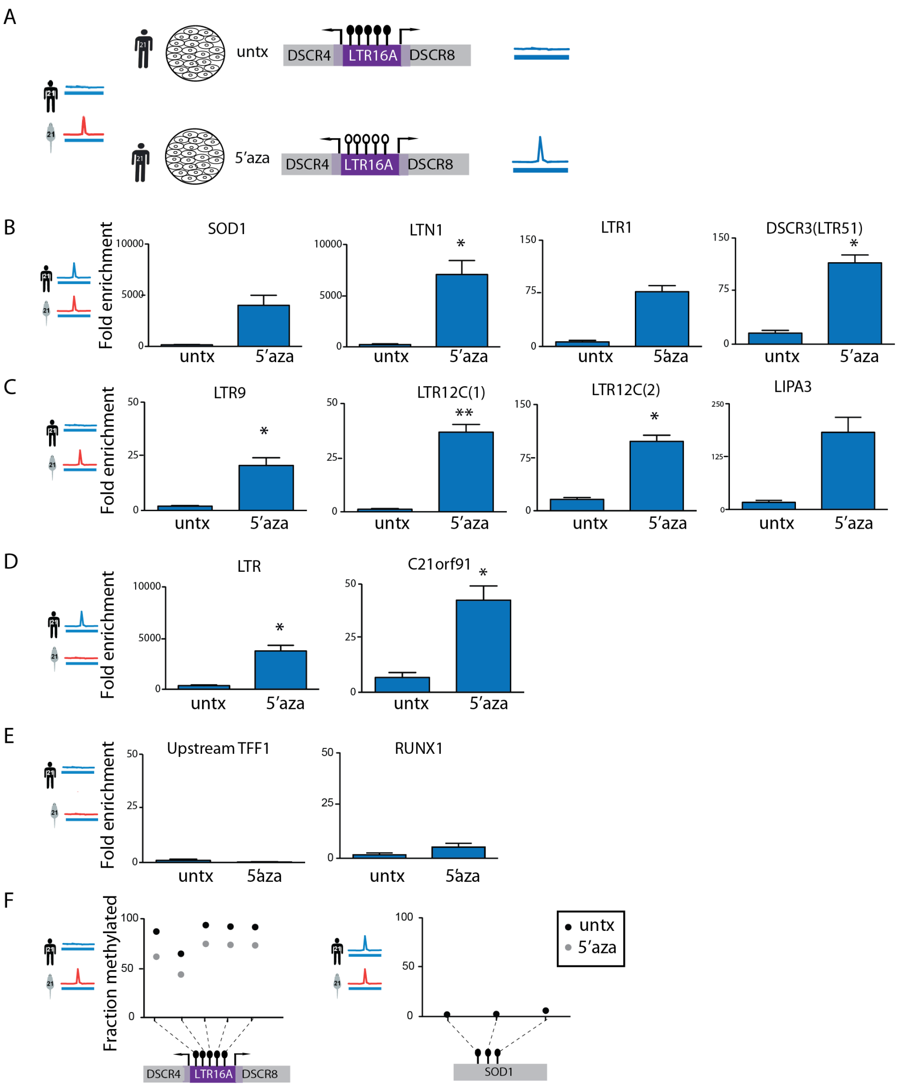

Figure S5.B: Abrogation of DNA methylation causes activation of latent human sites found in Tc1 mouse, related to Figure 5. HepG2 cells were treated with 1.5 uM 5-Aza-2-deoxycytidine (5-Aza-dCTP) for 48 hours prior to formaldehyde cross-linking. H3K4me3 ChIP was performed on treated and untreated cells and enrichment determined by qPCR at H3K4me3 sites identified by ChIP-seq analysis. A) Schematic of experimental design and hypothesis that upon DNA demethylation H3K4me3 sites identified exclusively in the Tc1 mouse can be re-directed to the same sites in a human cell line. B) H3K4me3 enrichment at sites with H3K4me3 in both Human and Tc1 liver as determined by ChIP-seq. C) H3K4me3 enrichment in Tc1-specific sites. D) H3K4me3 enrichment at Human-specific H3K4me3 sites. The *C21orf91* locus is deleted in the Tc1 mouse. E) H3K4me3 enrichment at two regions identified by the absence of a H3Kme3 ChIP-seq peak. F) DNA methylation analysis at a Tc1-specific and Shared H3K4me3 site in the presence and absence of 5-Aza-dCTP. qPCR results shown are representative of three independent experiments performed in triplicate where error bars show standard error of the mean. H3K4me3 regions and primer sequences used are shown in Table S1.E. (* p≤0.05 and ** p≤0.005, Student's t-test).

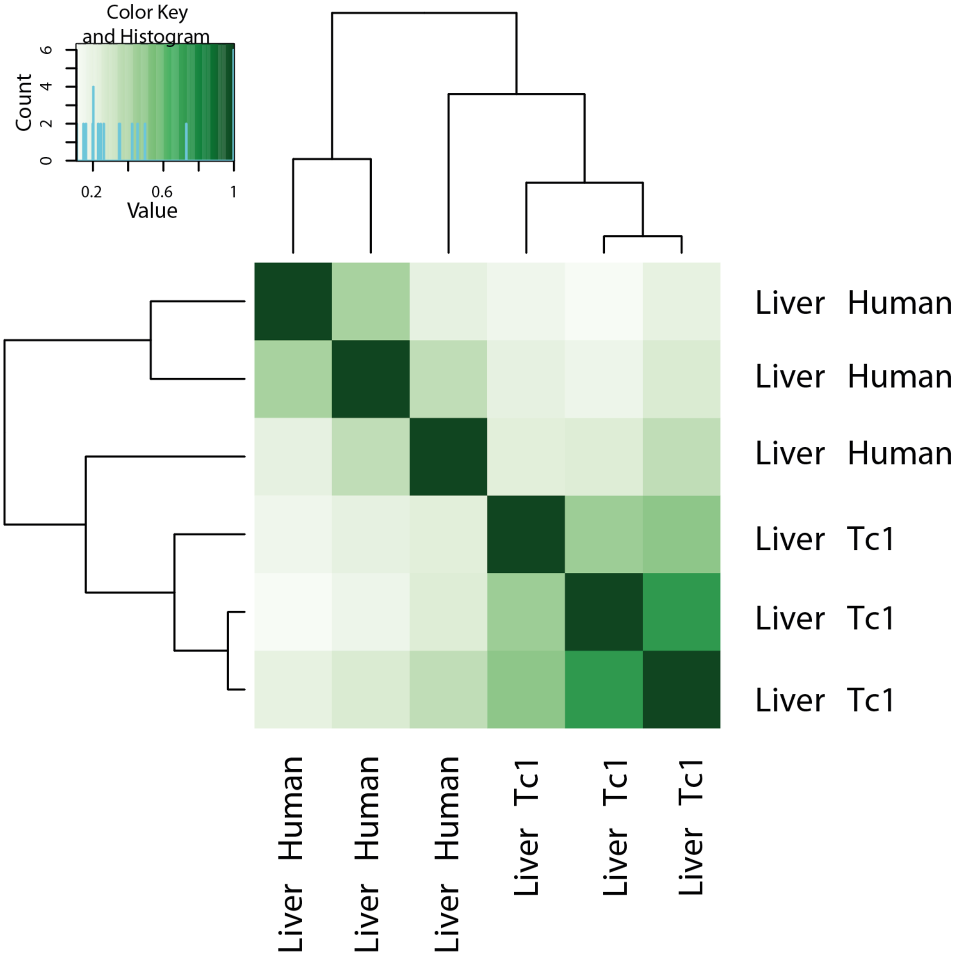

Figure S6.A: Human and Tc1 replicates cluster together, related to Figure 6. Three Tc1 and three Human H3K9me3 replicates cluster together. Correlation heatmap of log2 normalised read counts within 500 bp windows across a 37 Mb region including the H3K4me3 regions under study. Counts are reflective of Pearson correlation values between sample pairs and the heatmap generated using unsupervised hierarchical clustering.

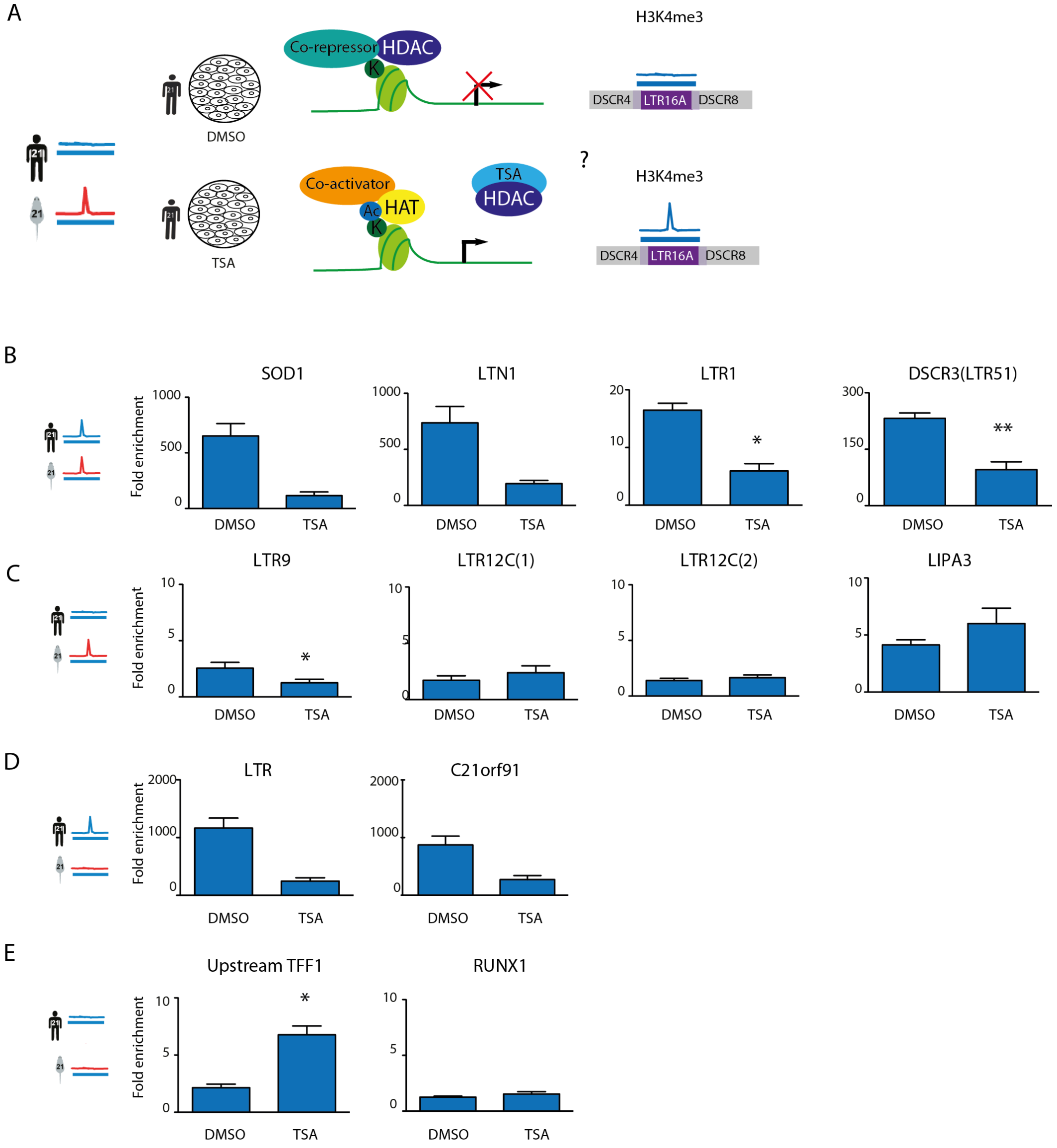

Figure S6.B: Inhibition of HDAC activity does not activate latent human sites found in Tc1 mouse, related to Figure 6. HepG2 cells were treated with 500 nM Trichostatin A (TSA) for 24 hours prior to formaldehyde cross-linking. H3K4me3 ChIP was performed on treated and untreated cells and enrichment determined by qPCR at H3K4me3 sites identified by ChIP-seq analysis. A) Schematic of experimental design and hypothesis that upon HDAC inhibition H3K4me3 sites identified exclusively in the Tc1 mouse can be re-directed to the same sites in a human cell line. B) H3K4me3 enrichment at sites with H3K4me3 in both Human and Tc1 liver as determined by ChIP-seq. C) H3K4me3 enrichment in Tc1-specific sites. D) H3K4me3 enrichment at Human-specific H3K4me3 sites. The *C21orf91* locus is deleted in the Tc1 mouse. E) H3K4me3 enrichment at two regions identified by the absence of a H3Kme3 ChIP-seq peak. qPCR results shown are representative of two independent experiments performed in triplicate where error bars show standard error of the mean. H3K4me3 regions and primer sequences used are shown in Table S1.E. (** p≤0.005, * p≤0.05, Student's t-test).

SUPPLEMENTAL TABLES

Table S1.A: ChIP-seq and input libraries have similar numbers of aligned reads, related to Figure 2. Summary of all human and Tc1 ChIP-seq and input libraries used. Human libraries were aligned to Hg18 and Tc1 mouse libraries to the Tc1 genome (mm9 + HsChr21 (hg18)). Only uniquely mapping reads were used in the analysis.

Tissue	Factor	Species	ChIP sample	Genome	Unique Aligned ChIP reads	Input sample	Unique Aligned Input reads	Sequencing	Read length (bp) (lane1,lane2)	Total peaks	All-Chr21 peaks	Tc1-chr21 peaks	Tc1:Hsa peaks
**Liver**	**H3K4me3**		DO270		13,455,439	DO253	9,140,987	SE	44				
			DO509		14,413,512	DO205	13,874,077	SE	41				
		Human	SE Pool	hg18	27,868,951		23,015,064	SE		21,887	220	172	
			DO756		31,590,479	DO758	31,506,342	PE	50				
			All Pool		59,459,430		54,521,406	PE + SE		25,250	268	211	
			DO211		21,678,250	DO20	6,706,256	SE	36,42				
			DO19		14,226,563	DO217	14,124,351	SE	36				
		Tc1	SE Pool	Tc1	35,904,813		20,830,607	SE		27,373	332	329	1.91
			DO755		35,337,585	DO757	35,464,057	PE	50				
			All Pool		71,242,398		56,294,664	PE + SE		26,299	339	335	1.59
**Kidney**	**H3K4me3**		DO815		42,269,966	DO819	36,717,640	PE	50				
		Human	DO947	hg18	17,316,705	DO948	19,881,671	SE	36				
			All Pool		59,586,671		56,599,311	PE + SE		20,848	224	182	
			DO813		49,423,538	DO817	46,698,207	PE	50				
		Tc1	DO965	Tc1	19,852,207	DO966	19,692,995	SE	36				
			All Pool		69,275,745		66,391,202	PE + SE		20,347	297	293	1.61
**Testes**	**H3K4me3**		DO912		25,961,487	DO915	24,303,961	SE	36				
		Human	DO1208	hg18	32,149,535	DO1229	21,894,113	SE	36				
			DO1209		30,160,599	DO1230	18,929,663	SE	36				
			All Pool		88,271,621		65,127,737	SE		39,607	503	418	
			DO812		54,838,831	DO816	48,548,327	PE	50				
		Tc1	DO679	Tc1	10,904,879	DO682	21,825,614	SE	36				
			All Pool		65,743,710		70,373,941	PE + SE		57,437	1,123	1101	2.63
**Brain**	**H3K4me3**		DO1816		24,586,630								
		Tc1	DO1819	Tc1	24,402,321								
			All pool		48,988,951	DO1856	24,287,694	SE	36	27,430	396	382	N/A
**Spleen**	**H3K4me3**		DO1818		27,553,779								
		Tc1	DO1821	Tc1	24,637,013								
			All pool		52,190,792	DO1858	23,946,033	SE	36	24,815	208	208	N/A
**Muscle**	**H3K4me3**		DO1854		17,500,797								
		Tc1	DO1855	Tc1	19,135,916								
			All pool		36,636,713	DO1859	22,188,63	SE	36	18,563	342	318	N/A
**Liver**	**H3K9me3**		DO1849		13,751,463	DO205	13,874,077	SE	36				
		Human	DO1851	hg18	24,684,550	DO758	31,506,342	SE	36				
			DO274		37,637,527	DO253	9,140,987	SE	36				
			All pool		76,073,540		54,521,406			N/A	N/A	N/A	N/A
			DO1848		11,994,661	DO217	14,124,351	SE	36				
		Tc1	DO1850	Tc1	12,550,533	DO20	6,706,256	SE	36				
			DO287		14,178,150	DO757	35,464,057	SE	44				
			All pool		38,723,344		56,294,664			N/A	N/A	N/A	N/A
**Liver**	**CEBPA**		DO204		18,177,941	DO203	8,313,049	SE	44,42				
		Human	DO206	hg18	19,114,189	DO205	13,874,077	SE	44,42				
			All Pool		37,292,130		22,187,126	SE		30,490	300	245	
			DO573		10,486,229	DO20	6,706,256	SE	36				
		Tc1	DO576	Tc1	11,137,650	DO217	14,124,351	SE	36				
			DO601		26,590,939			SE					
			All Pool		48,214,818		20,830,607	SE		39,668	197	197	0.80
**Liver**	**HNF4A**		DO505		15,425,128	DO205	13,874,077	SE	36				
		Human	DO199	hg18	13,966,010	DO137	7,240,796	SE	44,36				
			All Pool		29,391,138		21,114,873	SE		47,344	536	433	
			DO600		30,482,567	DO20	6,706,256	SE	42				
		Tc1	DO502	Tc1	31,934,367	DO217	14,124,351	SE	36,42				
			All Pool		62,416,934		20,830,607	SE		72,422	408	403	0.93
**Liver**	**CTCF**		DO778		24,145,336	DO648	10,056,754	SE	36				
		Human	DO506	hg18	30,059,956	DO205	13,874,077	SE	36,41				
			All Pool		54,205,292		23,930,831	SE		45,247	505	422	
			DO574		28,857,821	DO20	2,251,137	SE	36,41				
		Tc1	DO577	Tc1	30,720,936	DO217	14,124,351	SE	36,41				
			DO602		30,155,370			SE	36				
			All Pool		89,734,127		16,375,488	SE		49,056	278	278	0.66

Table S1.B: ChIP-seq experiments in wildtype mice do not cross-map to the transchromosomic human chromosome, related to Figure 2. A) Schematic representation of the experimental design. B) Wildtype mouse ChIP-seq data for H3K4me3, CEBPA, HNF4A and CTCF from C57BL/6J liver and testes and H3K4me3 from liver of a wild type Tc1 littermate that does not carry Tc1-HsChr21 (Tc0). Sequencing reads were aligned to the Tc1 genome (mm9 + HsChr21 (hg18)). C) Tc1 mouse ChIP-seq libraries aligned to the Tc1 genome.

A

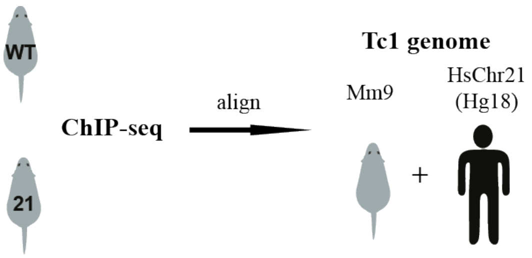

B

Wildtype mouse aligned to Tc1 genome

Library	Species	Tissue	ChIP	Read Length (bp)	Sequencing	Tc1 total reads	Mmus aligned reads	HsChr21 aligned reads	HsChr21 aligned reads (unique)	Unique HsChr21/ total aligned reads
do1316	Tc0	liver	H3K4me3	50	PE	162,647,964	162,416,820	231,144	68,792	4.E-04
do1240	C57BL/6J	liver	H3K4me3	36	SE	18,793,740	18,781,614	12,126	4,159	2.E-04
do843	C57BL/6J	liver	CEBPA	36	SE	22,273,035	22,268,437	4,598	1,360	6.E-05
do732	C57BL/6J	liver	HNF4A	36	SE	22,701,083	22,653,749	47,334	13,994	6.E-04
do781	C57BL/6J	liver	CTCF	36	SE	28,545,192	28,479,715	65,477	22,211	8.E-04
do1181	C57BL/6J	testes	H3K4me3	36	SE	28,864,021	28,833,517	30,504	10,229	4.E-04
do1319	Tc0	liver	Input	50	PE	154,881,570	154,876,930	4,640	861	6.E-06
do566	C57BL/6J	liver	Input	36	SE	16,896,634	16,893,910	2,724	783	5.E-05
do993	C57BL/6J	testes	Input	36	SE	26,039,594	26,038,239	1,355	217	8.E-06

C

Tc1 mouse aligned to Tc1 genome

Library	Species	Tissue	ChIP	Read Length (bp) lane1/lane2	Sequencing	Tc1 total reads	Mmus aligned reads	HsChr21 aligned reads	HsChr21 aligned reads (unique)	Unique HsChr21/ total aligned reads
do19/211/755	Tc1	liver	H3K4me3	36/36,42/50	SE/SE/PE	85,631,698	85,078,549	553,149	429,143	5.E-03
do573/576/601	Tc1	liver	CEBPA	36/36/36,42	SE	61,119,549	60,817,150	302,399	242,091	4.E-03
do502/600	Tc1	liver	HNF4A	36,42/42	SE	70,353,236	69,854,700	498,536	354,301	5.E-03
do574/577/602	Tc1	liver	CTCF	36,42/36,41/36	SE	102,935,990	102,484,841	451,149	385,822	4.E-03
do679/812	Tc1	testes	H3K4me3	36/50	SE/PE	74,144,070	73,369,307	774,763	524,281	7.E-03
do217/757	Tc1	liver	Input	36/50	SE/PE	64,634,313	64,283,666	774,763	524,281	8.E-03
do682/816	Tc1	testes	Input	36/50	SE/PE	88,764,347	88,244,979	519,368	474,118	5.E-03

Table S1.C: ChIP-seq regions of enrichment in wildtype mice do not overlap with regions of enrichment on Tc1-HsChr21, related to Figure 2. A) Numbers of histone/transcriptional regulator enriched regions in wildtype mice on the mouse genome and on Tc1-HsChr21, together with the number of regions overlapping the identified enriched regions in this study. B) Numbers of histone/transcriptional regulator enriched regions in Tc1 mice on the mouse genome and on Tc1-HsChr21.

A

Wildtype mouse regions of enrichment

Library	Species	Tissue	ChIP	Total enriched regions	Enriched regions Mmus	Enriched regions Tc1-HsChr21	Overlap of study peaks
do1316	Tc0	liver	H3K4me3	22,310	22,285	25	5
do1240	C57BL/6J	liver	H3K4me3	13,479	13,479	0	0
do843	C57BL/6J	liver	CEBPA	51,191	51,190	1	1
do781	C57BL/6J	liver	CTCF	56,262	56251	11	0
do732	C57BL/6J	liver	HNF4A	80,288	80,278	10	1
do1181	C57BL/6J	testes	H3K4me3	18,772	18,772	0	0

B

Tc1 mouse regions of enrichment

Library	Species	Tissue	ChIP	Total enriched regions	Enriched regions Mmus	Enriched regions Tc1-HsChr21	Overlap of study peaks
do19/211/755	Tc1	liver	H3K4me3	26,299	25,960	335	335
do573/576/601	Tc1	liver	CEBPA	39,668	39,471	197	197
do502/600	Tc1	liver	HNF4A	72,422	72,014	403	403
do574/577/602	Tc1	liver	CTCF	49,056	48,778	278	278
do679/812	Tc1	testes	H3K4me3	57,437	56,314	1101	1101

Table S1.D: Tc1-specfic transcription initiation sites are maintained following alignment of ChIP-seq data to a combined mouse-human genome, related to Figure 2. A) H3K4me3 ChIP-seq data in liver, kidney and testes was aligned to a composite human+mouse genome and regions of transcription initiation identified. This was done to verify that the Tc1-specific regions were not due to differences in sequence read mappability. These regions were compared to study regions identified following alignment to the Human or Tc1 genome. B) H3K4me3 ChIP-seq peak regions identified following Tc1 or Hsa genome alignment or Tc1 + Hsa genome alignment. The majority of Tc1-specific sites identified following alignment to the Tc1 genome are also found following alignment to the composite genome with many sites within repeats maintained. C) Transcriptional regulators as for B).

A

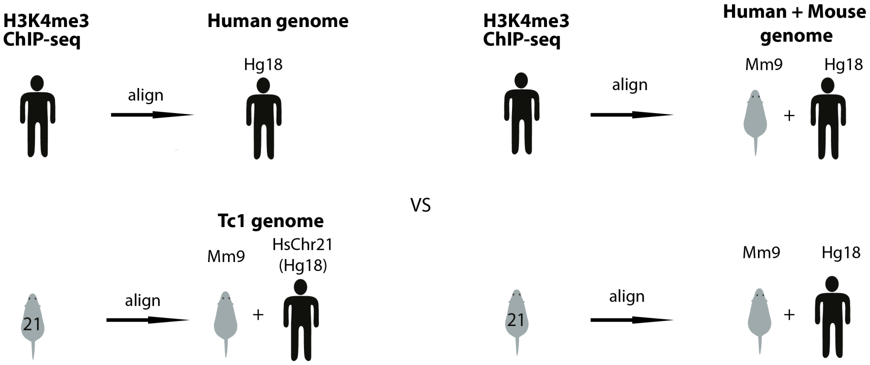

B H3K4me3

	H3K4me3 (Liver)			H3K4me3 (Kidney)			H3K4me3 (Testes)		
	Shared	Tc1	Human	Shared	Tc1	Human	Shared	Tc1	Human
Regions aligned to Tc1 or Hs	214	118	51	147	133	38	750	244	155
Regions aligned to Tc1 + Hs	203	100	35	141	125	38	649	185	132
Ratio of peak regions relative to shared (from Tc1 or Hs aligned)	1	0.55	0.24	1	0.9	0.25	1	0.33	0.21
Ratio of peak regions relative to shared (Tc1 + Hs aligned)	1	0.49	0.17	1	0.84	0.26	1	0.29	0.2
Tc1 or Hs vs Tc1+Hs overlapping regions	200	103	35	144	122	37	589	234	136
Tc1 or Hs vs Tc1 + Hs overlapping regions (same classification)	199	100	34	139	122	35	563	170	116
Fraction Tc1 or Hs regions in Tc1+Hs alignment	0.93	0.87	0.69	0.98	0.92	0.97	0.79	0.96	0.88
Fraction Tc1 or Hs regions in Tc1+Hs alignment (same classification)	0.93	0.85	0.67	0.95	0.92	0.92	0.75	0.70	0.75
Regions aligned to Tc1 or Hs with repeat at summit	25	56	15	17	64	9	432	112	102
Regions aligned to Tc1+Hs with repeat at summit	22	43	8	16	55	8	296	103	85
Fraction Tc1 or Hs repeat regions captured by Tc1+Hs alignment	0.88	0.77	0.53	0.94	0.86	0.89	0.69	0.92	0.83

C Transcriptional regulators

	CEBPA			HNF4A			CTCF		
	Shared	Tc1	Human	Shared	Tc1	Human	Shared	Tc1	Human
Regions aligned to Tc1 or Hs	203	48	66	340	112	140	358	39	50
Regions aligned to Tc1 + Hs	178	28	49	307	91	110	337	31	40
Ratio of peak regions relative to shared (from Tc1 or Hs aligned)	1	0.24	0.33	1	0.33	0.41	1	0.11	0.14
Ratio of peak regions relative to shared (Tc1 + Hs aligned)	1	0.16	0.28	1	0.30	0.36	1	0.09	0.12
Tc1/Hs vs Tc1+Hs genome overlapping regions	169	28	58	309	80	119	329	34	45
Tc1/Hs vs Tc1+Hs genome overlapping regions of same category	167	28	47	293	77	108	326	30	38
Fraction Tc1 or Hs regions in Tc1+Hs alignment	0.83	0.58	0.88	0.91	0.71	0.85	0.92	0.87	0.9
Fraction Tc1 or Hs regions in Tc1+Hs alignment (same classification)	0.82	0.58	0.71	0.86	0.69	0.77	0.91	0.77	0.76
Regions aligned to Tc1 or Hs with repeat at summit	63	26	26	73	56	42	77	15	10
Regions aligned to Tc1+Hs with repeat at summit	55	10	21	73	32	38	70	13	8
Fraction Tc1 or Hs repeat regions captured by Tc1+Hs alignment	0.87	0.38	0.81	1	0.57	0.90	0.91	0.87	0.80

Table S1.E: H3K4me3 ChIP-seq regions for qPCR validation, related to Figure 2. H3K4me3 regions (Hg18 co-ordinates) and primer sequences used for qPCR validation of identified ChIP-seq regions.

H3K4me3 Tc1/Hsa	Repeat/gene	H3K4me3 region	Forward primer sequence	Reverse primer sequence	Diffbind category
- / -	TFF2	chr21:42,644,115-42,645,554	AAGTTCATTTTCAGCCAACC	CTGTCAGGTCCACAGAATCC	None
- / -	RUNX1	chr21:35,704,599-35,704,671	GGAAATCACGCCTGGTAGAT	GGTATCTGCAGCCCTCTCTC	None
+ / +	SOD1	chr21:31,953,153-31,954,742	GCTGGTTTGCGTCGTAGTCT	GCCTTCTGCTCGAAATTGAT	Human
+ / +	LTN1	chr21:29,286,041-29,287,720	ACCGCTCTCAGGGACGCACA	AACCTGAGGGTGAGCGGGGG	Shared
+ / +	DSCR3(LTR51)	chr2: 37,551,868-37,553,017	ACCGATTGCGCAGGAGGCAC	GCAGGGCACGGAGACGCTTT	Shared
+ / +	LTR1	chr21:44,729,262-44,730,811	ATGCCGGCGGAAGAGCACAC	TCTCAGCCGACGGAGGAGCC	Shared
+ / -	LTR12C(1)	chr21:37,576,601-37,578,470	AACCCACGGAGGCGGGGTA	GCTGGCCCCGGGCAATAAGG	Tc1
+ / -	LIPA3	chr21:17,384,869-17,385,065	ACGAGCCAAAGCAGGGCAAGT	GGACCCTCCGAGCCAGGTGT	Tc1
+ / -	LTR9	chr21:38,414,987-38,416,146	AACATGGGTGGCAAAAAGAG	AGCGAAACTCCGACTCAAAA	Tc1
+ / -	LTR12C(2)	chr21:23,112,315-23,113,974	CCTTGGATGGTCGATAGGAC	GATTTCTCACCGGGCCTTAG	Tc1
- / +	LTR71B, AluSx, MIRb, MIR3	chr21:44,726,826-44,728,675	ATCGGACACCCGACCTCGCA	GAGGGAGGCACCGCAACGAG	Human
- / +	C21orf91	chr21:18,112,797-18,113,986	TCGCGGACCCAAGTCCGTCT	CCGACCCCCAGTCCCATCGT	None

Table S2.A: Tc1-specific sites in somatic tissue are enriched for repetitive elements, related to Figure 3. Fraction of ChIP-seq peak summits containing repetitive elements as classified by RepeatMasker.

Tissue	ChIP	Shared			Tc1-specific			Hsa-specific		
		Total	With repeat	Repeat fraction	Total	With repeat	Repeat fraction	Total	With repeat	Repeat fraction
Liver	HNF4A	337	80	**0.24**	112	56	**0.50**	140	42	**0.30**
Liver	CEBPA	203	63	**0.31**	48	26	**0.54**	65	25	**0.38**
Liver	CTCF	358	77	**0.22**	39	15	**0.38**	50	10	**0.20**
Liver	H3K4me3	214	25	**0.12**	118	56	**0.47**	51	14	**0.27**
Kidney	H3K4me3	147	17	**0.12**	133	64	**0.48**	37	9	**0.24**
Testes	H3K4me3	750	432	**0.58**	244	111	**0.45**	155	102	**0.66**

Table S2.B: Particular repeat types are enriched in Tc1-specific sites, related to Figure 3. Enrichment of repeat elements at H3K4me3, CEBPA, HNF4A and CTCF peak summits in Tc1-specific regions relative to Shared regions. Repeats enriched in the direction of Tc1-specific are shown in red while repeats enriched in the direction of Shared are shown in black. NA values represent no incidence of repeats in the Tc1-specific or Shared categories. p-values are calculated by the Chi-square test where p≤0.05 are represented in bold. Repeat classes that are significant in at least one data set are shown. A) Enrichment of repeat classes at ChIP-seq peak summits. B) Enrichment of repeat families at ChIP-seq peak summits. C) Enrichment of repeat names at ChIP-seq region summits. The complete statistical results are shown in File S2.

Repeat Class > Family > Name

A

Class

	Liver H3K4me3	Kidney H3K4me3	Testes H3K4me3	CEBPA	Liver HNF4A	CTCF
SINE	4.5E-01	5.5E-01	**2.3E-09**	**3.0E-06**	**4.6E-05**	6.6E-01
LINE	**1.3E-04**	**4.6E-03**	**3.8E-03**	2.0E-01	3.1E-01	8.2E-01
LTR	**8.2E-05**	**7.1E-06**	9.1E-02	8.5E-01	5.1E-02	**4.7E-02**
DNA	7.6E-01	9.6E-01	1.3E-01	5.0E-01	1.0E+00	6.6E-01
Satellite	8.2E-02	1.1E-01	5.5E-01	**4.4E-03**	1.0E-01	1.8E-01
Other	**1.0E-02**	**2.9E-02**	**1.8E-04**	NA	NA	NA
Simple_repeat	4.9E-01	9.6E-01	9.8E-01	NA	**3.7E-03**	NA
Low_complexity	3.3E-01	3.5E-01	8.5E-01	NA	NA	1.8E-01
All_Repeats	**7.6E-09**	**6.7E-09**	2.7E-01	**1.1E-02**	**1.8E-05**	3.1E-01

B

Family

	Liver H3K4me3	Kidney H3K4me3	Testes H3K4me3	CEBPA	Liver HNF4A	CTCF
Alu	4.5E-01	2.1E-01	**3.9E-09**	**5.8E-07**	**2.7E-07**	8.6E-01
L1	**9.1E-04**	**1.6E-02**	**2.7E-03**	3.5E-01	1.0E+00	6.7E-01
ERV1	**1.0E-05**	**1.5E-05**	3.2E-01	8.5E-01	5.1E-01	7.3E-02
MaLR	4.9E-01	9.3E-01	2.9E-01	8.8E-01	3.4E-01	**2.0E-02**
Other	**1.0E-02**	**2.9E-02**	**1.8E-04**	NA	NA	NA
Simple_repeat	4.9E-01	9.6E-01	9.8E-01	NA	**3.7E-03**	NA
telo	8.2E-02	1.1E-01	5.5E-01	**4.4E-03**	1.0E-01	1.8E-01

C

Name

	Liver H3K4me3	Kidney H3K4me3	Testes H3K4me3	CEBPA	Liver HNF4A	CTCF
AluJb	7.6E-01	NA	5.5E-01	**2.1E-03**	8.1E-02	4.7E-01
AluJo	NA	NA	3.0E-01	**4.6E-04**	1.0E-01	NA
AluSg	7.5E-01	NA	5.7E-01	1.7E-01	**1.9E-02**	NA
AluSq	7.6E-01	NA	5.7E-01	**4.4E-02**	5.6E-01	NA
AluSx	7.6E-01	NA	9.1E-01	3.8E-01	**1.1E-02**	4.7E-01
AluY	**3.7E-03**	2.1E-01	**1.2E-08**	7.3E-01	7.4E-01	NA
SVA	**1.0E-02**	**2.9E-02**	**1.8E-04**	NA	NA	NA
L1PA2	**2.9E-02**	1.1E-01	**4.0E-03**	NA	NA	NA
LTR12C	**9.1E-04**	**1.6E-02**	7.6E-01	NA	5.6E-01	1.8E-01
LTR12D	**2.9E-02**	1.1E-01	5.7E-01	NA	NA	**1.9E-03**
LTR17	NA	NA	1.4E-02	NA	NA	NA
TAR1	2.4E-01	4.3E-01	9.9E-01	**4.4E-03**	1.0E-01	1.8E-01

Table S2.C: Repeat regions captured by H3K4me3 are reflective of the distribution of repeat classes across HsChr21 and the human genome, related to Figure 3. Total numbers and percentages of repeat class instances in the human genome, on HsChr21, and within our H3K4me3 regions is shown. There is a similar percentage of LINE, LTR and SINE repeat classes on HsChr21.

Repeat Class	Total Hg18 repeats Class	Number repeats Tc1-Chr21 regions	Number repeats H3K4me3 regions	Percent Hg18 repeats	Percent Tc1_Chr21 repeats	Percent H3K4me3 region repeats
DNA	392529	3846	82	9.2	9.2	7.9
LINE	1406996	13597	234	33.0	32.5	22.4
LTR	656486	9682	258	15.4	23.1	24.7
Other	3425	20	5	0.1	0.0	0.5
RNA	718	5	0	0.0	0.0	0.0
SINE	1783780	14519	447	41.8	34.7	42.9
Satellite	8895	103	14	0.2	0.2	1.3
Unknown	929	19	0	0.0	0.0	0.0
rRNA	1753	20	1	0.0	0.0	0.1
scRNA	1399	6	0	0.0	0.0	0.0
snRNA	4340	43	2	0.1	0.1	0.2
srpRNA	958	4	0	0.0	0.0	0.0
tRNA	1956	13	0	0.0	0.0	0.0
Total	4264164	41877	1043	100.0	100.0	100.0

Table S3: The majority of liver Tc1-specific H3K4 trimethylated regions are observed across a panel of somatic tissues, while testes regions are largely unique, related to Figure 3. Categorised liver and testes H3K4me3 regions were overlapped with all H3K4me3 regions identified in Tc1 kidney, brain, muscle and spleen. Numbers and fractions of H3K4me3 regions shared between liver (A) and testes (B) and each of the additional somatic tissues.

A Liver centric

		# in kidney	Fraction in kidney	# in testes	Fraction in testes	# in brain	Fraction in brain	# in muscle	Fraction in muscle	# in spleen	Fraction in spleen
Liver H3K4me3 categories	Liver total (384)	258	0.67	287	0.75	265	0.69	223	0.58	187	0.49
	Liver Tc1-specific (118)	95	**0.81**	107	**0.91**	100	**0.85**	87	**0.74**	70	**0.59**
	Liver Shared (214)	148	0.69	161	0.75	150	0.70	124	0.58	110	0.51
	Liver Human-specific (51)	14	0.27	19	0.37	15	0.29	12	0.24	7	0.14
Liver H3K4me3 categories with repeat at summit	Liver Tc1-specific (56)	48	**0.86**	54	**0.96**	51	**0.91**	47	**0.84**	38	**0.68**
	Liver Shared (25)	14	0.56	19	0.76	12	0.48	13	0.52	12	0.48
	Liver Human-specific (14)	2	0.14	4	0.29	2	0.14	2	0.14	1	0.07
Liver Tc1-specific H3K4me3 with significant repeat types at summit	Liver Tc1-specific with AluY (8)	5	**0.63**	8	**1.00**	7	**0.88**	5	**0.63**	1	**0.13**
	Liver Tc1-specific with SVA (5)	5	**1.00**	5	**1.00**	5	**1.00**	5	**1.00**	2	**0.40**
	Liver Tc1-specific with L1PA2 (5)	4	**0.80**	5	**1.00**	5	**1.00**	5	**1.00**	3	**0.60**
	Liver Tc1-specific with LTR12C (10)	10	**1.00**	10	**1.00**	10	**1.00**	10	**1.00**	10	**1.00**
	Liver Tc1-specific with LTR12D (5)	5	**1.00**	5	**1.00**	5	**1.00**	5	**1.00**	5	**1.00**
Liver Shared H3K4me3 with significant repeat types at summit	Liver Shared with AluY (0)	-	-	-	-	-	-	-	-	-	-
	Liver Shared with SVA (0)	-	-	-	-	-	-	-	-	-	-
	Liver Shared with L1PA2 (0)	-	-	-	-	-	-	-	-	-	-
	Liver Shared with LTR12C (1)	1	1.00	1	1.00		0.00	1	1.00	1	1
	Liver Shared with LTR12D (0)	-	-	-	-			-			
Liver excluded		72		812		122		95		21	

B Testes centric

		# in liver	Fraction in liver	# in kidney	Fraction in kidney	# in brain	Fraction in brain	# in muscle	Fraction in muscle	# in spleen	Fraction in spleen
Testes H3K4me3 categories	Testes total (1149)	272	0.24	252	0.22	300	0.26	248	0.22	185	0.16
	Testes Tc1-specific (244)	55	0.23	42	0.17	61	0.25	52	0.21	23	0.09
	Testes Shared (750)	206	**0.27**	201	**0.27**	221	**0.29**	183	**0.24**	159	**0.21**
	Testes Human-specific (155)	23	0.15	51	0.33	18	0.12	13	0.08	3	0.02
Testes H3K4me3 categories with repeat at summit	Testes Tc1-specific (112)	11	0.10	24	0.21	26	0.23	36	0.32	17	0.15
	Testes Shared (432)	43	0.10	44	0.10	51	0.12	47	0.11	32	0.07
	Testes Human-specific (102)	4	0.04	3	0.03	6	0.06	4	0.04	0	0.00
Testes Tc1-specific H3K4me3 with significant repeat types at summit	Testes Tc1-specifc with AluY (15)	2	0.13	1	0.07	1	0.07	2	0.13	0	0.00
	Testes Tc1-specific with SVA (9)	4	0.44	5	0.56	5	0.56	7	0.78	2	0.22
	Tetses Tc1-specific with L1PA2 (6)	5	0.83	5	0.83	6	1.00	6	1.00	3	0.50
	Testes Tc1-specific with LTR12C (5)	3	0.60	3	0.60	3	0.60	4	0.80	3	0.60
	Testes Tc1-specific with LTR12D (1)	0	0.00	0	0.00	0	0.00	0	0.00	0	0.00
Testes Shared H3K4me3 with significant repeat types at summit	Testes Shared with AluY (212)	6	**0.03**	5	**0.02**	6	**0.03**	6	**0.03**	1	**0.00**
	Testes Shared with SVA (3)	1	0.33	1	0.33	1	0.33	2	0.67	1	0.33
	Testes Shared with L1PA2 (1)	0	0.00	0	0.00	0	0.00	0	0.00	0	0.00
	Testes Shared with LTR12C (9)	8	0.89	8	0.89	8	0.89	8	0.89	8	0.89
	Testes Shared with LTR12D (5)	5	1.00	5	1.00	5	1.00	5	1.00	5	1.00
Testes excluded		65		42		86		70		23	

Table S4: Pol III binds to AluY elements in Tc1 testes and liver. ChIP-seq was performed in duplicate for Tc1 mouse testes and Tc1 mouse liver. Peaks were called using CCAT3.0 with the provided TF_config file and the number of significant peaks reported. All testes Pol III peaks had an FDR ≤ 0.05. For liver, all significant peaks are shown with the number of peaks with an FDR ≤ 0.05 indicated in parentheses. Due to the low mappability of the AluY elements bound by Pol III, both uniquely mapping reads (as was done for all other analyses in this manuscript) and all mapping reads were analysed. Although these results show more significant Pol III occupancy at AluY regions on Tc1-HsChr21 in testes, the low mappability of AluY prevents robust comparisons to be made between tissues.

	**Testes**		**Liver**	
	All reads	Unique reads	All reads	Unique reads
Pol III Peaks	21189	15170	4853 (990)	1309 (436)
Pol III peaks on Tc1-Chr21	3044	147	2695 (229)	78 (3)
Tc1-Chr21 Pol III summit overlapping AluY	602	1	521 (17)	1 (0)
Tc1-Chr21 Pol III peaks overlapping H3K4me3	661	113	156 (31)	19 (1)
Tc1-Chr21 Pol III summit overlapping AluY in H3K4me3 regions	165	0	13 (0)	1 (0)
Number AluY on Tc1-chr21 in H3K4me3 regions	500	500	41	41
Number AluY on Tc1-chr21	1679	1679	1679	1679

Table S5: A subset of Tc1-specifc transcriptional regulator binding sites are associated with H3K4me3, related to Figure 4. Tc1-specific and Shared transcriptional regulator sites were overlapped with H3K4 trimethylation events in liver.

	**Tc1-specific**				**Shared**			
	**Category**	**CTCF**	**CEBPA**	**HNF4A**	**Category**	**CTCF**	**CEBPA**	**HNF4A**
Transcriptional regulator sites	Total	39	48	112	Total	358	203	337
Transcriptional regulator sites with H3K4me3	Any H3K4me3	22	19	51	Any H3K4me3	90	79	153
	Tc1-specific H3K4me3	13	13	32	Shared H3K4me3	66	55	119
	Fraction overlapping any H3K4me3	0.56	0.40	0.46	Fraction overlapping any H3K4me3	0.25	0.39	0.45
	Fraction overlapping Tc1-specific H3K4me3	**0.33**	**0.27**	**0.29**	Fraction overlapping Shared H3K4me3	**0.18**	**0.27**	**0.35**
Transcriptional regulator sites at significantly enriched repeats	Any repeat	15	26	56	Any repeat	77	63	80
	LTRs	4	0	5	LTRs	12	4	3
	Alus	0	16	18	Alus	3	15	8
H3K4me3 at transcriptional regulator sites at significantly enriched repeats	LTRs Overlap Tc1-specific H3K4me3	4	-	4	LTRs Overlap Shared H3K4me3	0	1	1
	Fraction LTR-bound sites with H3K4me3	**1.00**	-	0.80	Fraction LTR-bound sites with H3K4me3	-	0.25	0.33
	Alu Overlap Tc1-specific H3K4me3	0	2	3	Alu Overlap Shared H3K4me3	-	2	1
	Fraction Alu-bound sites with H3K4me3	-	**0.13**	**0.17**	Fraction Alu-bound sites with H3K4me3	-	**0.13**	**0.13**

Table S6.A: Tc1-specific H3K4me3 regions are hypomethylated in the Tc1 mouse, related to Figure 5. Percentage DNA methylation at each interrogated CpG site in livers from three different Tc1 and human individuals. CpG sites used for bisulphite pyrosequencing experiments were identified on the basis of presence (+) or absence (-) of the H3K4me3 mark in human and Tc1 tissue. CpG chromosome coordinates shown from hg18. The average percentage DNA methylation of CpG sites present on the Illumina Infinium Human Methylation450K beadarrays are shown in the last two columns. Regions plotted in Figure 5 are highlighted in grey. The H3K4me3 regions and primer sequences used are shown in Table S6.B and were identified as described in methods.

H3K4me3 Tc1/Hsa	Repeat/gene	CpG region assayed	CpG site	Tc1 (% methylation)			Human (% methylation)			Diffbind Category	DNA methylation array Tc1 ave (%)	DNA methylation array Human ave (%)
-/-	AluSc (WRB)	chr21:39,673,427-39,673,516	1	68	62	71	49	48	61	Shared	-	-
			2	79	69	84	70	72	76		-	-
			3	81	70	83	67	70	76		-	-
-/-	DSCAM	chr21:40,472,564-40,472,631	1	68	62	71	49	48	61	None	-	-
			2	79	69	84	70	72	76		-	-
			3	81	70	83	67	70	76		-	-
+/+	MRPL39	chr21:25,901,292-25,901,327	1	1	1	14	1	1	2	Shared	-	-
			2	1	1	13	1	1	1		-	-
			3	2	1	1	1	2	2		-	-
+/+	ADAMTS1	chr21:27,140,651-27,140,676	1	4	9	2	4	5	3	Human	8	5
			2	2	2	1	1	2	2		-	-
			3	6	3	2	2	5	5		-	-
			4	7	2	1	3	4	3		-	-
+/+	U2AF1	chr21:43,399,954-43,399,979	1	6	1	3	1	1	2	Shared	-	-
			2	9	5	4	4	4	3		-	-
			3	2	2	3	2	2	2		-	-
+/+	SOD1	chr21:31,954,946-31,954,986	1	3	3	4	2	2	4	Human	-	-
			2	5	2	4	3	3	5		-	-
			3	21	18	15	15	14	20		-	-
+/+	CLDN14	chr21:36,773,705-36,773,724	1	15	29	24	32	37	35	Human	-	-
			2	14	30	28	31	45	37		-	-
+/-	LIPA3	chr21:17,384,676-17,384,820	1	16	10	16	68	71	63	Tc1	-	-
			2	29	36	22	96	93	96		-	-
			3	16	18	7	85	87	84		-	-
			4	12	14	6	68	72	66		-	-
			5	17	19	6	82	83	82		-	-
			6	18	19	11	80	81	78		-	-
+/-	LTR16A	chr21:38,415,394-38,415,422	1	49	43	57	100	100	100	Tc1	30	58
			2	34	27	41	90	88	91		37	59
			3	32	32	39	93	94	93		23	86
			4	30	24	31	93	95	95		-	-
			5	24	24	34	90	92	93		-	-
+/-	SH3BGR	chr21:39,745,592-39,745,596	1	10	8	12	78	80	85	Tc1	35	69
			2	7	5	10	76	77	82		-	-
			3	6	5	7	66	63	66		-	-
			4	6	6	7	66	62	71		-	-
+/-	ITGB2	chr21:45,132,719-45,132,735	1	15	15	18	66	68	69	Tc1	-	-
			2	21	20	28	76	80	77		-	-
			3	22	20	22	59	63	61		-	-
			4	39	21	33	89	88	89		-	-
+/-	SH3BGR	chr21:39,745,749-39,745,793	1	10	8	12	78	80	85	Tc1	-	-
			2	7	5	10	76	77	82		-	-
			3	6	5	7	66	63	66		-	-
			4	6	6	7	66	62	71		-	-
+/-	LTR12D	chr21:31,382,886-31,382,916	1	49	43	57	100	100	100	Tc1	-	-
			2	34	27	41	90	88	91		-	-
			3	32	32	39	93	94	93		-	-
			4	30	24	31	93	95	95		-	-
			5	24	24	34	90	92	93		-	-
+/-	LIPA4	chr21:28,971,293-28,971,312	1	2	2	2	81	86	83	Tc1	-	-
			2	4	3	2	89	88	89		-	-
			3	5	3	2	82	86	87		-	-
			4	8	4	2	90	91	92		-	-
-/+	CRYAA	chr21:43,464,233-43,464,251	1	64	55	52	52	56	53	None	-	-
			2	77	62	55	53	60	56		-	-
			3	93	79	82	62	68	67		-	-
-/+	CLDN14	chr21:36,836,912-36,836,928	1	4	10	10	57	56	55	Human	11	53
			2	9	6	10	55	56	59		32	55
			3	4	10	10	53	49	49		-	-
-/+	LTR71B, AluSx Mirb, MIR3	chr21:44,727,877-44,727,924	1	75	76	100	33	37	44	Human	-	-
			2	75	85	90	38	41	53		-	-
			3	75	84	75	45	47	58		-	-
			4	85	92	91	36	38	50		-	-
			5	78	84	87	33	31	39		-	-
-/+	COL6A1	chr21:46,226,246-46,226,276	1	7	10	9	6	6	5	Human	-	-
			2	3	3	2	5	3	3		-	-
			3	16	8	10	8	8	5		-	-
			4	5	7	7	6	4	4		-	-
			5	5	4	4	3	3	3		-	-
-/+	DNMT3L	chr21:44,493,195-44,493,222	1	50	47	59	44	54	59	Human	-	-
			2	66	56	74	75	81	87		-	-
			3	51	36	60	56	58	68		-	-
-/+	C21orf34	chr21:16,714,107-16,714,121	1	57	59	47	8	9	10	Human	-	-
			2	70	66	60	9	10	12		-	-
			3	76	74	59	8	9	10		-	-

Table S6.B: Regions interrogated for DNA methylation analysis, related to Figure 5. Genome co-ordinates of H3K4me3 regions and primer sequences used in the DNA pyrosequencing analysis. Regions plotted in Figure 5 are highlighted in grey.

H3K4me3 Tc1/Hsa	Repeat/gene	H3K4me3 region	Forward primer sequence	Reverse primer sequence	Sequencing primer sequence
- / -	Alu (WRB)	chr21:39,673,160-39,673,560	ATGTTGGTTAGGTTGGTTTTAAT	AAAAAATCCCAACTCAAATATATA	TTAATTTTTTGATTTTATGATTTG
- / -	DSCAM	chr21:40,472,068-40,472,887	AGTTTTGGGTATGGAAATTTG	ACTTAAATACAAAAACAAACCTTT	AGTGTTTTTAAAGTAAGATGTAG
+/+	MRPL39	chr21:25,900,929-25,902,340	TTTTTTGGGAAGATATGGATAGAT	AAAAAAAAAATTTCTTAATCCCTC	GGAAGATATGGATAGATGTG
+/+	ADAMTS1	chr21:27,137,225-27,141,165	GGTTTTATATTTTTGGAATGGGTG	CTTAAACCCCTCCCACTTTT	GGAAGGGAAAAGGTTATA
+/+	U2AF1	chr21:43,399,696-43,400,852	ATGGGTTTATAGGGGTATTAAAGG	ACTTAACCAACCTCTTACCTATTT	GGTTTATAGGGGTATTAAAGGTAT
+/+	SOD1	chr21:31,953,821-31,955,115	ATTTGTTTTAGTGTAGGGTGAGTA	ATAAAAACTCCAAATCTTCTAATT	TTTAAAAGTGATTTTGATGTG
+/+	CLDN14	chr21:36,772,777-36,774,260	GGTAGGGTTTAAGGGTTTTTAAAATAGAT	CCAAACCACCTCCTCTCAT	TTTTGTTTAGGTTTTTTGTTAG
+/-	LIPA3	chr21:17,384,603-17,385,519	GGGATATAGTTTAGTTTGTTATAG	AAATAAAAAATACAAAAATCACCCA	AGGGGGGAGGAGTTA
+/-	LTR16A	chr21:38,414,850-38,415,936	AAGAGTGAGGAGATTAGTTTTTAGATA	CTCTTTTTACCACCCATATTTACAA	GTGGAGTTTAGAGATTG
+/-	SH3BGR	chr21:39,745,529-39,746,071	GGTGTATAGTATAGTTTGGTTTTT	CTCCAAAAAAACAACAAAAACATCT	GGTTTTTTAGGGATTGT
+/-	ITGB2	chr21:45,132,368-45,133,996	GGGTTATTTTTGGGGATTTGTAAT	TCCCCTCCTCACCTAACC	AAGTTTTGTTTTATGAGGTATTTA
+/-	SH3BGR	chr21:39,745,529-39,746,071	GTTGTTTTTTGGAGAGATAGAGTT	CCCAACACACCCCTATCAA	TTGGAGAGATAGAGTTTTTTA
+/-	LTR12D	chr21:31,382,508-31,383,076	GTGAAGGGTTTTTGGAGTAGG	AAACCACTACACCCCATATAATAA	GTTTTTGGAGTAGGGAT
+/-	LIPA4	chr21:28,970,636-28,971,785	GAGATAAAGGGTTGGTGAATATTG	ATATAAAACCCTCAAAAACCTCT	TGATTTTATAAGTTGATATTTAAG
- / +	CRYAA	chr21:43463446-43464641	TTAGAAAGAATTTTAGGAAGTGGG	ACAAACTAAAAACCCCCTTATTCC	ATTTTATTTGAGGAAGTTAGTT
- / +	CLDN14	chr21:36,835,697-36,837,036	GAGGTTGGTAGTGAAAGAAATT	ACCCACAAAAATAATTCCACTCT	TTTAGGAGAGTTGGGGGA
- / +	LTR71B, AluSx, Mirb, MIR3	chr21:44,727,273-44,728,601	GTTAGGGTTGTGAGTAAAAGTGTA	AAACTAAATAATCTCCAAAATCCC	GGGAGGGGAGAGGAT
- / +	COL6A1	chr21:46226152-46227096	GTTGTTGTAGGTTTGTTGGATAG	AACCCCCAATCTACAATTCC	AACCACCACTCACCC
- / +	DNMT3L	chr21:44494065-44495051	GGGAGGTGGGGAGGTTATA	TCCCCTTAAAAAACTTCTCCAACA	GTTTGGGGGTTGTAG
- / +	C21orf34	chr21:16713425-16715746	AGTTGGGTAGTGAGATTGT	ATTTCCCTTCTAAATAACACTTTC	TGTTTAAATTTTAAAGGAATATTG
	M13R 5' biotin tag			CGCCAGGGTTTTCCCAGTCACGAC 5' rev or *5' fwd	

Table S6.C: Tc1-specific H3K4me3 events are generally associated with lower levels of DNA methylation in Tc1 mouse compared to human, related to Figure 5. CpG methylation levels in Tc1 and human liver and testes DNA were determined using an Illumina Infinium Human Methylation450k beadarray. CpGs with significantly detectable signal in Tc0 mice were excluded from the analysis leaving 3174 CpG sites to interrogate across the chromosome. DNA methylation was classified into Methylated (>80% DNA methylation), Partially methylated (20-80% DNA methylation) and Unmethylated (<20% DNA methylation). Total numbers of CpG sites within 100 bp of the peak summit are shown for each methylation category.

Factor	Tissue	Methylation staus	Shared		Tc1-specifc		Human-specific		All CpGs	
			Human	Tc1	Human	Tc1	Human	Tc1	Human	Tc1
H3K4me3	Liver	Methylated	3	1	8	1	1	0	1581	1172
		Partially methylated	5	25	5	10	0	3	674	1531
		Unmethylated	35	17	2	4	2	0	919	471
H3K4me3	Testes	Methylated	19	15	11	2	2	3	1577	1215
		Partially methylated	22	58	1	7	0	1	669	1648
		Unmethylated	51	19	0	3	3	1	928	311
CTCF	Liver	Methylated	0	1	0	0	1	1	1581	1172
		Partially methylated	10	25	2	2	0	1	674	1531
		Unmethylated	31	15	3	3	1	0	919	471
CEBPA	Liver	Methylated	3	3	0	0	0	1	1581	1172
		Partially methylated	13	21	1	3	4	3	674	1531
		Unmethylated	15	7	2	0	0	0	919	471
HNF4A	Liver	Methylated	1	2	2	0	0	0	1581	1172
		Partially methylated	10	23	2	8	3	6	674	1531
		Unmethylated	30	16	5	1	7	4	919	471

EXPERIMENTAL PROCEDURES

ChIP-seq

Antibodies

The following antibodies were used: H3K4me3 rabbit polyclonal antibody (ab8580/ab1012, Abcam), H3K4me3 mouse monoclonal antibody (CMA304) and H3K9me3 mouse monoclonal antibody (clone 2F3), were kind gifts from Dr Hiroshi Kimura (Chandra et al., 2012; Kimura et al., 2008; Matsui et al., 2010) Pol II (ab5408, Abcam), and Pol III 1900 from Dr Robert J. White (Fairley et al., 2003). The following antibodies were used in the previously published datasets: CEBPA (sc-9314, Santa Cruz Biotech) and HNF4A (ARP31946, Aviva Biosystems) (Schmidt et al., 2010) and CTCF (07729, Millipore) (Schmidt et al., 2012).

Library preparation

The immunoprecipitated material was end-repaired, A-tailed, ligated to single- or paired-end sequencing adapters, amplified by 18-cycles of PCR and size selected (200 - 300 bp) followed by single/paired-end (SE/PE) sequencing on an Illumina Genome Analyzer II or HiSeq according to the manufacturer's instructions as previously described (Schmidt et al., 2009).

Differential binding analysis

Enrichment analysis was performed as in (Ross-Innes et al., 2012) using the DiffBind R/Bioconductor package (version 1.0) (Stark, R. & Brown, G. D. DiffBind: differential binding analysis of ChIP-seq peak data. Bioconductor (2011) http://bioconductor.org/packages/release/bioc/html/DiffBind.html) for analysis of differential binding. Sequencing data were normalized by the TMM method using the effective library size after subtracting control reads; normalization and statistical tests were computed using the edgeR package (Robinson et al., 2010). Analyses were performed using both the shared region's coordinates that corresponded to the Tc1 or Human bound regions (these were nearly identical and all shared coordinates and peak summits shown in the manuscript are presented from the Tc1 perspective). To be

considered differentially bound (Tc1-specific or Human-specific) the normalized number of reads between the two species was required to be at least four- fold different and have an FDR of less than 0.10. Data for H3K4me3, CTCF, HNF4A and CEBPA were analysed independently using peak called regions, whereas log2 normalised read counts were used for the analysis of the inherently diffuse H3K9me3 ChIP-seq data.

Heatmap generation

To generate heatmaps of raw ChIP-seq data, the summit of the pooled SE and PE human H3K4me3/transcription factor enriched regions on human chromosome 21 (Shared and Human-specific) and the summit of the Tc1-chromosome 21 regions uniquely enriched for H3K4me3 (Tc1-specific) were used as targets to centre each window. Each window was divided into 100 bins of 100 bp in size. An enrichment value was assigned to each bin by counting the number of sequencing reads in that bin and subtracting the number of reads in the same bin of an input library. Each dataset was normalized to the same number of sequencing reads. Data were visualized with Treeview (Page, 1996).

Validation of H3K4me3 ChIP-seq enrichment

Real-time PCR analysis was used to validate the H3K4me3 regions identified by ChIP-seq. Primers were designed to ChIP-seq peak regions using Primer-BLAST web-based tool (NCBI). Diluted ChIP and input libraries were amplified using Power SYBR® Green PCR Master Mix (Applied Biosystems) with the following two-step PCR cycling conditions: 10 min at 95°C followed by 40 cycles of 15 seconds at 95°C and 30 s at 60°C on a ABI7900HT machine (Applied Biosystems). Enrichment was calculated using the $\Delta\Delta C_T$ method (Livak and Schmittgen, 2001) with values normalised to a control region showing no enrichment and to the corresponding input DNA.

RNA-seq

Gene expression and H3K4me3 association analysis

H3K4me3 binding events were categorized as Shared or Tc1-specific, associated with its closest transcript and the log expression of Tc1 transcripts relative to human calculated. Statistical significance between categories was determined using the one-sided Mann-Whitney U test.

Repeat analysis

Statistics for repeat assignments

In order to determine the enrichment of repeat classes within H3K4me3 or transcriptional regulator bound regions, the enrichment of repeat classes in the Tc1-specific regions relative to the Shared regions was determined by the number of cases of a particular repeat element occurring at the peak summit with the Shared regions being based on a Tc1-centric perspective. p-values were calculated using a Chi-square test on a 2x2 contingency table.

Repeat age analysis

H3K4me3 or transcriptional regulator enriched regions were filtered, retaining those whose summit was embedded in a repeat element. The age of individual bound repeat elements was estimated by dividing the number of substitutions from the consensus (milliDiv column in the UCSC-obtained RepeatMasker tracks) by the mutation rate estimated for mammalian species (2.2×10^{-9} per base pair per year) (Kumar and Subramanian, 2002). The distribution of all values for Shared and Tc1-specific categories were displayed as box plots and the distributions compared with the Wilcoxon rank-sum test. A random background was generated by reshuffling (1000 repetitions) of the category assignment (Shared, Tc1-specific, Human-specific).

DNA methylation analysis

Locus-specific DNA methylation assays

DNA was extracted from 10-100 mg human and Tc1 liver extracted using the Easy-DNA Kit (Invitrogen). 1 μg of DNA was treated with a CT conversion reagent using the EZ DNA Methylation-Gold Kit^{TM} (Zymo) according to manufacturer's instructions. Forward, reverse and sequencing primers were designed to the predicted bisulphite treated DNA sequence of interest using PyroMark Assay Design 2.0 (Qiagen). A 3' biotinylated M13 primer and a 5'-M13-tagged forward primer were used in a nested PCR reaction in order to obtain a biotin-labelled product. Primers were designed around Tc1-specific, Shared or Human-specific H3K4me3 sites containing 3-6 CpG sites within ~50 bp within a total amplicon length of 80 -200 bp. Forward and reverse primers were used to amplify the region of interest with 2x Thermomix High Performance PCR Mix (ABgene). The cycling conditions were 95°C for 10 min; followed by 24 cycles of 95°C for 30 s, 56°C for 30 s, 72°C for 1 min and a final elongation step at 72°C for 5 min. A second round of PCR was performed with the M13 biotinylated reverse primer and the assay specific forward primer. Second round PCR was performed at 95°C for 10 min followed by 39 cycles of 95°C for 30 s, 56°C for 30 s, 72°C for 1 min followed by a final extension at 72°C for 10 min. Products were electrophoresed on a 2% agarose gel to ensure specificity of amplification. The amplified PCR product was purified using Streptavidin-coated Sepharose beads (GE Healthcare) with shaking at 800-1000 rpm for 30 minutes at room temperature followed by final purification using the Vacuum Prep Tool (Biotage). Purified DNA was incubated at 80°C for 3 minutes to denature the template and allow annealing of the sequencing primer during cooling to room temperature. The PCR products, with annealed sequencing primers, were sequenced on a PyroMark Q96 MD pyrosequencer (Qiagen) using PyroMark Gold SQA Reagents (Qiagen) according to the maunfacturer's instructions. The methylation status of the PCR products was calculated on the ratio of cytosine to converted thymidine nucleotides using Pyro Q-CpG^{TM} software (Qiagen).

REFERENCES

Chandra, T., Kirschner, K., Thuret, J.Y., Pope, B.D., Ryba, T., Newman, S., Ahmed, K., Samarajiwa, S.A., Salama, R., Carroll, T.*, et al.* (2012). Independence of Repressive Histone Marks and Chromatin Compaction during Senescent Heterochromatic Layer Formation. Mol Cell *47*, 203-214.

Fairley, J.A., Scott, P.H., and White, R.J. (2003). TFIIIB is phosphorylated, disrupted and selectively released from tRNA promoters during mitosis in vivo. EMBO J *22*, 5841-5850.

Kimura, H., Hayashi-Takanaka, Y., Goto, Y., Takizawa, N., and Nozaki, N. (2008). The organization of histone H3 modifications as revealed by a panel of specific monoclonal antibodies. Cell Struct Funct *33*, 61-73.

Kumar, S., and Subramanian, S. (2002). Mutation rates in mammalian genomes. Proc Natl Acad Sci U S A *99*, 803-808.

Livak, K.J., and Schmittgen, T.D. (2001). Analysis of relative gene expression data using real-time quantitative PCR and the 2(-Delta Delta C(T)) Method. Methods *25*, 402-408.

Matsui, T., Leung, D., Miyashita, H., Maksakova, I.A., Miyachi, H., Kimura, H., Tachibana, M., Lorincz, M.C., and Shinkai, Y. (2010). Proviral silencing in embryonic stem cells requires the histone methyltransferase ESET. Nature *464*, 927-931.

Page, R.D. (1996). TreeView: an application to display phylogenetic trees on personal computers. Comput Appl Biosci *12*, 357-358.

Robinson, M.D., McCarthy, D.J., and Smyth, G.K. (2010). edgeR: a Bioconductor package for differential expression analysis of digital gene expression data. Bioinformatics *26*, 139-140.

Ross-Innes, C.S., Stark, R., Teschendorff, A.E., Holmes, K.A., Ali, H.R., Dunning, M.J., Brown, G.D., Gojis, O., Ellis, I.O., Green, A.R.*, et al.* (2012). Differential oestrogen receptor binding is associated with clinical outcome in breast cancer. Nature *481*, 389-393.

Schmidt, D., Schwalie, P.C., Wilson, M.D., Ballester, B., Goncalves, A., Kutter, C., Brown, G.D., Marshall, A., Flicek, P., and Odom, D.T. (2012). Waves of Retrotransposon Expansion Remodel Genome Organization and CTCF Binding in Multiple Mammalian Lineages. Cell.

Schmidt, D., Wilson, M.D., Ballester, B., Schwalie, P.C., Brown, G.D., Marshall, A., Kutter, C., Watt, S., Martinez-Jimenez, C.P., Mackay, S.*, et al.* (2010). Five-vertebrate ChIP-seq reveals the evolutionary dynamics of transcription factor binding. Science *328*, 1036-1040.

Schmidt, D., Wilson, M.D., Spyrou, C., Brown, G.D., Hadfield, J., and Odom, D.T. (2009). ChIP-seq: using high-throughput sequencing to discover protein-DNA interactions. Methods *48*, 240-248.
